# Exploring potential mechanism of ciwujia tablets for insomnia by UPLC-Q-TOF-MS/MS, network pharmacology, and experimental validation

**DOI:** 10.3389/fphar.2022.990996

**Published:** 2022-08-30

**Authors:** Hongda Liu, Le Yang, Chunlei Wan, Zhineng Li, Guangli Yan, Ying Han, Hui Sun, Xijun Wang

**Affiliations:** ^1^ National Chinmedomics Research Center, National TCM Key Laboratory of Serum Pharmacochemistry, Metabolomics Laboratory, Department of Pharmaceutical Analysis, Heilongjiang University of Chinese Medicine, Harbin, China; ^2^ State Key Laboratory of Dampness Syndrome of Chinese Medicine, The Second Affiliated Hospital of Guangzhou University of Chinese Medicine, Guangzhou, China; ^3^ State Key Laboratory of Quality Research in Chinese Medicine, Macau University of Science and Technology, Taipa, Macau SAR, China

**Keywords:** insomnia, nervous system disease, ciwujia tablet, UPLC-Q-TOF-MS/MS, neurotransmitter

## Abstract

Insomnia, whether chronic or intermittent, is a common central nervous system disease. Ciwujia Tablet (CWT) is a well-known traditional Chinese medicine (TCM) made from the extract of *Eleutherococcus senticosus* (Rupr. & Maxim.) Maxim. This medication is commonly used for treating insomnia in China, but the lack of in-depth research focused on the chemical ingredients of CWT creates a gap in knowledge regarding its effective constituents against insomnia. Considering that the therapeutic material basis, targets, and pathways related to this drug have not been fully investigated by scholars in the field, the focus of this study is on identifying the chemical ingredients or structural characteristics of CWT by the UPLC-Q-TOF-MS/MS technique. Besides, concepts of network pharmacology were also used to investigate the targets and pathways of CWT. An insomnia rat model was established by intraperitoneal injection of p-chlorophenylalanine, and the results were verified through various experiments. A total of 46 ingredients were identified in CWT, such as eleutheroside B, eleutheroside E, isofraxidin, and chlorogenic acid. Among them, 17 ingredients with good solubility, favorable gastrointestinal absorption, and high bioavailability were selected for network pharmacological analysis. It was concluded that CWT participated in the regulation of neurotransmitter levels, modulation of ion transport, neurotransmitter receptor activity, synaptic transmission, dopaminergic transmission and other essential processes. Results from the animal experiments showed that CWT can increase the content of inhibitory neurotransmitters 5-HT and GABA in the brain, reduce the synthesis of excitatory escalating transmitters DA and NE, shorten the sleep latency and prolong the sleep duration of insomnia rats. Furthermore, CWT could significantly alleviate the symptoms of insomnia in model rats. Identifying the chemical ingredients of CWT in this experiment is of great significance for exploring its potential curative effects, which provides a solid basis for further understanding the therapeutic value of this medication.

## 1 Introduction

Acute or chronic insomnia is a common central nervous system disorder (Dopheide, 2020; Ai et al., 2021) that can negatively impact people’s daily work and life routines (Dopheide, 2020; Sagherian et al., 2021). Besides, it can change the autonomic nervous system and increase sympathetic nerve activity (Grimaldi et al., 2021), causing long-term brain fatigue that would increase energy expenditure. Studies in this field indicate that insomnia is correlated with the morbidity and mortality of cardiovascular diseases (CVDs), including hypertension (HTN), coronary heart disease (CHD), and heart failure (HF) (Javaheri and Redline, 2017; Tobaldini et al., 2017). The pathogenesis may be related to increased sympathetic activity, dysregulation of the hypothalamic-pituitary axis, and increased inflammation (Javaheri and Redline, 2017). Moreover, insomnia is considered a risk factor for HTN (Cappuccio and Miller, 2017; Bathgate and Fernandez-Mendoza, 2018), cognitive impairment (Irwin and Vitiello, 2019; Zhang et al., 2021), obesity, and diabetes (Knutson et al., 2017; Mokhlesi et al., 2019).

Ciwujia Tablets (CWT) are made from the extract of *Eleutherococcus senticosus* (Rupr. & Maxim.) Maxim (ES) (Ch.P. 2020), specifically its dry root and rhizome or stem (Ch.P. 2020). This plant is mainly distributed in China, North Korea, Japan, and Russia (Kawano et al., 2021), and related studies have confirmed that it has multiple pharmacological benefits, such as anti-neuroinflammation, antibacterial, anti-cancer (Kim et al., 2018; Kawano et al., 2021), anti-inflammatory (Lee et al., 2021), anti-oxidation (Kim et al., 2015), anti-AChE, and anti-BuChE features (Li et al., 2021). Additionally, it has positive effects on the cardiovascular (Jin et al., 2020), central nervous, and immune systems (Jia et al., 2021). According to the latest *Chinese Pharmacopoeia*, CWT is mainly used for treating insomnia in clinical scenarios (Ch.P. 2020). This drug is considered safe for use and has fewer side effects when administered in normal doses. However, since the chemical composition of CWT has not been fully clarified, its pharmacodynamic material basis and favorable mechanisms in the treatment of insomnia remain unclear. Therefore, properly identifying the chemical ingredients or structural characteristics of CWT is an urgent demand and may solidify the essential knowledge experts rely on to further explore the pharmacodynamic material of this herb.

The composition of natural medicines and different active ingredients have attracted the attention of many modern scholars in recent years (Goni et al., 2021; Uddin Chy et al., 2021). Some natural medicines and their active ingredients have been studied in detail, and some studies have revealed some positive effects they can have in treating many serious diseases and viral infections (Küpeli Akkol et al., 2020; Fernández et al., 2021), including the most recent COVID-19 (Islam et al., 2021; Tallei et al., 2021). However, research on traditional Chinese medicine (TCM) and natural medicines is hampered by some complications regarding complex ingredients and lack of reference compounds. Due to its high selectivity and sensitivity, the UPLC-Q-TOF-MS/MS technique is considered an appropriate means to accurately analyze the ingredients of CWT. Recently, this technique has been used to analyze TCM and other natural medicines (Bo et al., 2021; Cheng et al., 2021; El-Sabagh et al., 2021; Lin et al., 2021), revealing details of unknown components that are considered groundbreaking discoveries in the field. In this experiment, the UPLC-Q-TOF-MS/MS technique was adopted to analyze the ingredients of CWT, and the methodology was combined with other network pharmacology techniques used to examine the potential targets and metabolic pathways of CWT ingredients in the treatment of insomnia. This mixed research design can provide novel insights into the active ingredients of CWT and offer a basis for identifying its pharmacodynamic materials.

## 2 Materials and methods

### 2.1 UPLC-Q-TOF-MS/MS analysis of ciwujia tablet’s chemical ingredients

#### 2.1.1 Materials and reagents

The pure compounds of eleutheroside B, eleutheroside E, chlorogenic acid, and isofraxidin were used as reference standards (purity ≥ 98%). Eleutheroside B (Cat.No. wkq19041510), eleutheroside E (Cat.No. wkq19010212), and isofraxidin (Cat.No. wkq19070305) were provided by Sichuan Weikeqi Biological Technology Co., Ltd. (Sichuan, China), and the chlorogenic acid (Cat.No. 110753–200413) used in the experiment was originally produced by China National Institute for the Control of Pharmaceutical and Biological Products (Beijing, China). The MS-grade acetonitrile and methanol came from Thermo Fisher Scientific (Waltham, MA, United States), and Ciwujia Tablets (Batch No. 20190701) were produced by Heilongjiang Wusulijiang Pharmaceutical Co., Ltd. (Harbin, China). Estazolam tablets (Batch No. 200619) used in the experiment are a product of Shandong Xinyi Pharmaceutical Co., Ltd. (Dezhou, China). P-chlorophenylalanine (PCPA) was provided by Shanghai Macklin Biochemical Co., Ltd. (Shanghai, China) and phosphate buffered saline (PBS; 0.01 mol/L, pH 7.4) by Shanghai Yuanye Biotechnology Co., Ltd. (Shanghai, China). Normal saline (0.9% NaCl) came from Harbin Sanlian Pharmaceutical Co., Ltd. (Harbin, China) and formic acid from Aladdin Biochemical Technology Co., Ltd. (Shanghai, China). The distilled water is a product of Guangzhou Watson’s Food & Beverage Co., Ltd. (Guangzhou, China), and rat brain tissues 5-HT, GABA, DA, and NE ELISA kits were purchased from the Nanjing Jiancheng Bioengineering Institute (Nanjing, China).

#### 2.1.2 Sample preparation and standard solution

The first step of the experiment was preparing the sample solution by grounding some CWTs into fine powder after the sugar-coating film was removed. The preparation methods were optimized before the experiment to determine the best conditions to conduct the necessary steps. Approximately 1.0 g of powder was accurately weighed and ultrasonicated for 45 min with 10 ml of 50% methanol-water (v/v). Then, an additional 50% of methanol-water (v/v) was added to supplement the lost weight. The mixture was centrifuged at 13000 rpm for 5 min to obtain the supernatant.

Subsequently, the standard solution was prepared. All the standard ingredients were individually dissolved in 50% methanol-water (v/v) (approximately 1 mg/ml). The stock solution of each standard ingredient was further diluted with 50% methanol-water (v/v) until it reached the appropriate concentration. All solutions were stored at -20°C in the dark and then placed under ambient conditions before use.

#### 2.1.3 UPLC-Q-TOF-MS/MS qualitative analysis

In this study, the SCIEX Ekspert ultraLC UHPLC system was selected as the ultra-high performance liquid chromatograph. The AB SCIEX Triple TOF 5600 + mass spectrometry system equipped with an ESI source and an APCI source (AB Sciex, Foster City, CA, United States) was selected as the mass spectrometer. Analyst TF1.6 was adopted for data acquisition and Peakview 1.2 was used for data processing.

The chromatographic detection conditions involved a chromatographic separation performed at 40°C through a Waters Acquity UPLC HSS T3 Column (2.1 mm × 100 mm, 1.8 μm) with the mobile phase composed of acetonitrile (A) and water (B) (both including 0.1% formic acid, v/v). The elution procedure was performed as follows: 1) 0–5 in, 1%–18% A; 2) 5–20 min, 18%–55% A; 3) 20–25 min, 55%–70% A; 4) 25–28 min, 70%–99% A; 5) 28–30 min, 99% A. The flow rate was kept at 0.2 ml/min, and the volume of the injected sample was 2 μl.

The ideal mass spectrometry detection conditions involved the adoption of the ESI mode and positive and negative ion source voltages of 5000 V and -4500 V, respectively. The ion source temperature was 600°C and the cracking voltage (DP) was 80 V and −80 V, respectively. The collision energy (CE) was 35 eV and −35 eV, respectively; the collision energy expansion (CES) was 15 eV and −15 eV, respectively; the atomization gas (Gas1) was 50 psi; the auxiliary gas (Gas2) was 50 psi; the curtain gas was 35 psi. Furthermore, a full MS scan (*m/z* 50–1,200) was acquired, followed by top 10 information-dependent acquisition (IDA) MS/MS scans (*m/z* 50–1,200). In terms of the criteria for the IDA precursor selection, the top 10 intense peaks and response values larger than 100 CPS were selected. The dynamic background subtraction (DBS) was initiated after that.

#### 2.1.4 Ingredient identification and analysis

The UPLC-Q-TOF-MS/MS detection system was used to detect the sample solution of CWT. The possible element composition and a molecular formula of the ingredients in CWT were deduced according to the mass spectrum data. The ingredients of CWT could be finally identified by combining the standard products with the web databases of the Encyclopedia of Traditional Chinese Medicine (ETCM) (Xu et al., 2019) (http://www.tcmip.cn/ETCM/index.php/Home/Index/), the Traditional Chinese Medicine Integrated Database (Huang et al., 2018) (http://www.megabionet.org/tcmid/), and different relevant articles. The fragmentation pathway of the ingredients was inferred by secondary ion fragments.

### 2.2 Exploration of the mechanism of ciwujia tablet in the treatment of insomnia by network pharmacology

#### 2.2.1 Screening of intersection targets between ciwujia tablet chemical components and insomnia

CWT ingredients were screened using the swissADME web tool (Daina and Zoete, 2016; Daina et al., 2017) (http://www.swissadme.ch/index.php) based on the ingredient identification results. Then, the solubility class, GI absorption, and bioavailability score were selected as screening indicators. The ingredients with good solubility, good gastrointestinal absorption, and high bioavailability were selected as the research objects. The targets of these chemical components were collected according to the probability score. The targets related to insomnia were collected through the online databases of Genecards (Fishilevich et al., 2017) (https://www.genecards.org/) and OMIM (Amberger et al., 2015) (https://omim.org/). Finally, a diagram of the CWT component targets and the insomnia targets was plotted through the Venny platform to obtain the intersection targets.

#### 2.2.2 Construction of the protein-protein interaction network diagram of key anti-insomnia targets

The intersection genes of CWT component targets and insomnia were imported into the String (Szklarczyk et al., 2019) (https://string-db.org/) database for protein-protein interaction (PPI) analysis. Then, the PPI network of active CWT components and insomnia was constructed. Cystoscape 3.8.0 tool was adopted to obtain core targets based on degree values.

#### 2.2.3 Gene ontology and KEGG Pathway Enrichment Analysis

The obtained core targets were analyzed by GO and KEGG through the Metascape data platform (https://metascape.org/). The most important pathways were screened for animal experiment verification.

### 2.3 Animal experiment verification

#### 2.3.1 Animal grouping and treatment

Male Sprague Dawley (SD) rats (weight 220 ± 20 g) were purchased from Liaoning Changsheng Biotechnology Co., Ltd. (Liaoning, China) (NO: 2020-0001). SD rats were randomly divided into a control group (C; *n* = 20) and a model group (M; *n* = 60). Except for group C, rats in group M were intraperitoneally injected with p-chlorophenylalanine (PCPA) [400mg/(kg∙d)] for 4 consecutive days to construct an insomnia rat model. Those in group C were injected with the same amount of normal saline. After establishing the model, 10 rats were selected from groups C and M, respectively, for model evaluation. The remaining rats from the group M were randomly divided into other five groups: 1) a model group (M; *n* = 10); 2) a positive drug group [E, *n* = 10; estazolam tablet 0.2 mg/(kg∙d)], 3) a CWT high-dose group [CWT-H, *n* = 10; 600 mg/(kg∙d)], 4) a CWT middle-dose group [CWT-M, *n* = 10; 300 mg/(kg∙d)], and 5) a CWT low-dose group [CWT-L, *n* = 10; 150 mg/(kg∙d)]. The dosage of CWT for rats was determined according to results from previous studies, experimental pharmacological methods, and the (Reagan-Shaw et al., 2008; Lee et al., 2012; Chinese Pharmacopoeia Comission, 2020; Wang et al., 2021). According to the *Chinese Pharmacopoeia*, the ideal dosage for adults is 6 tablets per day (each tablet contains 250 mg of ES extract): 1.5g/(60  kg∙d). The body surface area (BSA) conversion was used to determine the final dosage, which ensured the research significance of this design. During the treatment process, the rats in groups C and M received the same dose of water. The drug was administered once a day for 7 consecutive days (Ma et al., 2013; Hua et al., 2021).

#### 2.3.2 Sleep test and related tests

The sleep test was performed on the fourth day after the establishment of the insomnia model and the seventh day of treatment. First, the rats in each group were intraperitoneally injected with sodium pentobarbital (30 mg/kg). The intraperitoneal injection time, sleep-onset time, and wake-up time were recorded, and the sleep latency and sleep duration were calculated. Then, the effect of CWT on sleep of insomnia rats was observed. At the same time, the corresponding test and verification were performed according to the key pathways obtained from the GO and KEGG analyses. The specific flow of the experimental design is shown in Supplementary Figure S1. All experimental procedures were approved by the Animal Protection and Ethics Committee of Heilongjiang University of Chinese Medicine (Approval No. 2022030602), and all experiments were carried out in accordance with the Declaration of Helsinki.

## 3 Results

### 3.1 Collection of UPLC-Q-TOF-MS/MS chromatogram of ciwujia tablet

The test solutions were prepared according to guidelines explained in Section 2.1.2. Before testing the samples, the solvents for extracting CWT were optimized. Water, 25% methanol, 50% methanol, 75% methanol, and 100% methanol were used as extracting solvents. More and higher response values of chromatographic peaks were obtained when 50% methanol was used as an extracting solvent. The ultrasonic extraction time was also investigated. The ultrasonic extraction time was also investigated at 15, 30, 45 and 60 min. After the experiments, it was concluded that the number of chromatographic peaks increased, and the response value of chromatographic peaks was higher under the ultrasonic extraction condition of 45 min.

**FIGURE 1:**
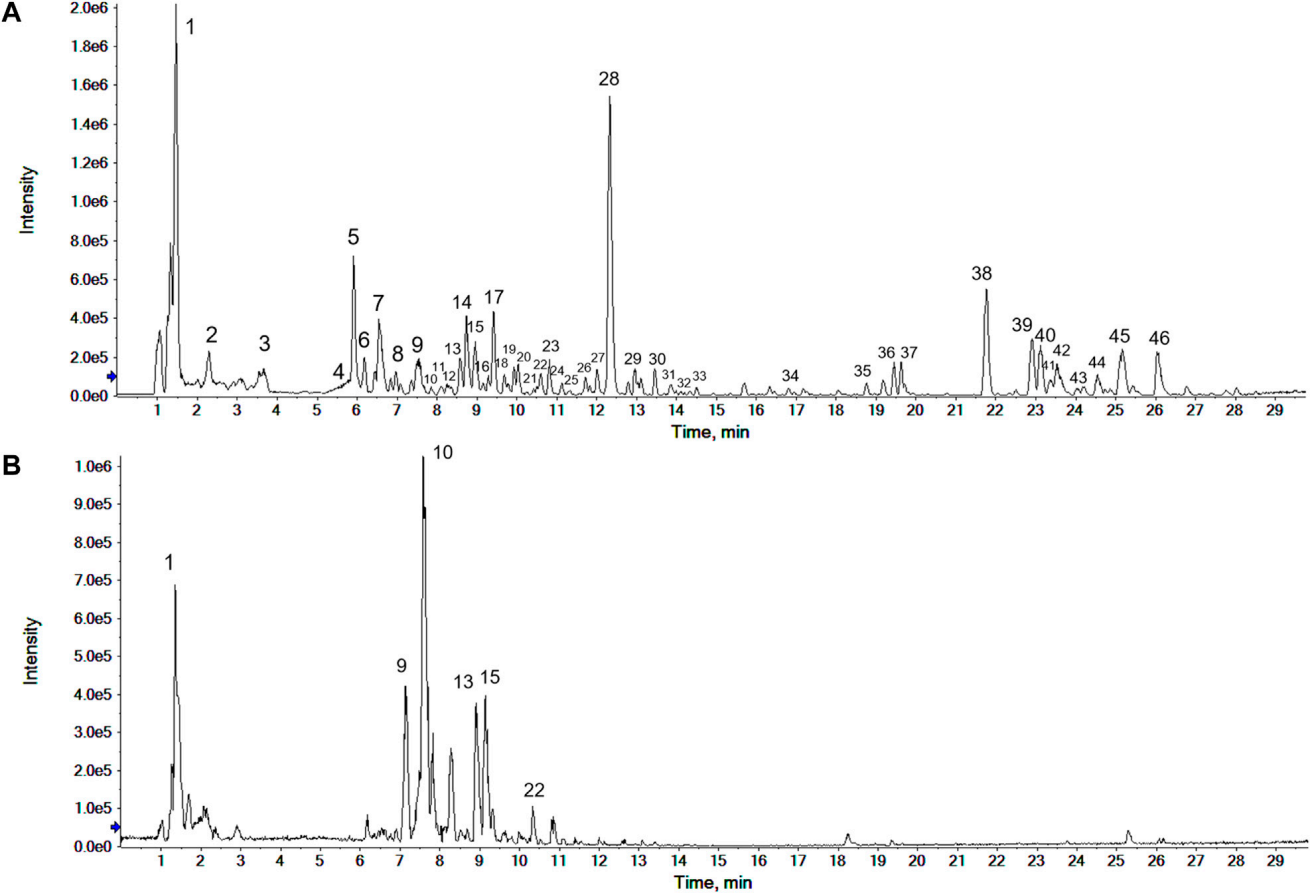
Base peak chromatogram (BPC) of CWT test solution in the positive **(A)** and the negative ion modes **(B)**.

The sample of CWT was analyzed in the positive and negative ion modes according to the conditions mentioned in Section 2.1.3. The base peak chromatogram (BPC) is shown in Figure 1. The compound in the extraction solution produced the [M + H]^+^ peak or [M + Na]^+^ peak in the positive ion mode and the [M-H]^-^ peak in the negative ion mode. The chemical ingredients of CWT in both ion modes were analyzed after identifying the peaks.

### 3.2 Ingredient identification analysis

According to the analysis methods outlined in Section 2.1.4 and relevant articles (Song et al., 2012; Sun et al., 2016; Wang et al., 2019; Jin et al., 2020), 46 chemical ingredients were identified in the total ion flow chromatogram of CWT. The details of these ingredients are listed in Table 1. The analysis results showed that the ingredients detected in the negative ion mode could also be detected in the positive one. Therefore, only the research data and major outcomes from the positive ion mode are provided in this paper. The analysis results of the negative model are shown in Supplementary Table S1. In addition, the structures of various chemicals were characterized. The representative identification process is detailed in the following sections.

**TABLE 1:** Ingredients identified in CWT based on UPLC-Q-TOF MS/MS.

No	Peak appearance time (min)	Ion species	Molecular formula	Theoretical value (*m/z*)	Value (*m/z*)	Deviation ΔPPM	Ion fragment (*m/z*)	Ingredient name
1	1.46	[M + H]^+^	C_24_H_28_O_4_	381.0878	381.0815	2.3	*m/z* 381.0815 [M + H]^+^	Clausarin
							*m/z* 353.0857 [M + H-C_2_H_4_]^+^	
							*m/z* 339.0796 [M + H-C_3_H_6_]^+^	
2	2.28	[M + H]^+^	C_11_H_21_NO_7_	280.1325	280.1385	3.8	*m/z* 280.1385 [M + H]^+^	Fructosylvaline
							*m/z* 262.1295 [M + H-H_2_O]^+^	
3	3.56	[M + H]^+^	C_9_H=NO_3_	182.0755	182.0794	3.2	*m/z* 363.1518 [2M + H]^+^	Tyrosine
							*m/z* 182.0794 [M + H]^+^	
4	5.87	[M + H]^+^	C_10_H_13_N_5_O_4_	268.1074	268.1026	3.9	*m/z* 268.1026 [M + H]^+^	Adenosine
							*m/z* 136.0604 [M + H-C_5_H_8_O_4_]^+^	
5	5.91	[M + H]^+^	C_17_H_9_NO_3_	276.1483	276.1418	1.3	*m/z* 276.1418 [M + H]^+^	Liriodenine
							*m/z* 268.1052 [M + H-H_2_O]^+^	
							*m/z* 136.0623 [M + H-H_2_O]^+^	
6	6.18	[M + H]^+^	C_30_H_50_O	427.1905	427.1821	4.8	*m/z* 427.1821 [M + H]^+^	Friedelin
7	6.44	[M + H]^+^	C_9_H_11_NO_2_	166.0873	166.0842	1.9	*m/z* 166.0842 [M + H]^+^	Phenylalanine
							*m/z* 120.0797 [M + H-HCOOH]^+^	
							*m/z* 103.0532 [M + H-HCOOH-NH_3_]^+^	
8	7.09	[M + H]^+^	C_16_H_16_O_8_	337.1725	337.1693	1.6	*m/z* 337.1693 [M + H]^+^	5-*O*-Caffeoylshikimic acid
							*m/z* 163.0393 [M + H-C_7_H_10_O_5_]^+^	
							*m/z* 145.0288 [M + H-C_7_H_12_O_6_]^+^	
9	7.54	[M + H]^+^	C_16_H_18_O_9_	355.0983	355.0974	0.7	*m/z* 355.0974 [M + H]^+^	Neochlorogenic acid
							*m/z* 163.0372 [M + H-C_7_H_12_O_6_]	
							*m/z* 145.0264 [M + H-C_7_H_14_O_7_]	
10	7.66	[M + H]^+^	C_26_H_26_O_12_	531.1705	531.1667	1.9	*m/z* 531.1667 [M + H]^+^	1-caffeoyl-5-feruloylquinic acid
							*m/z* 369.1141 [M + H-C_9_H_6_O_3_]^+^	
							*m/z* 177.0527 [M + H-C_16_H_17_O_9_]^+^	
11	7.85	[M + H]^+^	C_7_H_12_O_6_	193.0801	193.0866	2.7	*m/z* 193.0866 [M + H]^+^	Quinic acid
							*m/z* 147.0868 [M + H-CH_2_O_2_]^+^	
12	8.10	[M + H]^+^	C_16_H_18_O_8_	339.1097	339.1075	2.3	*m/z* 339.1075 [M + H]^+^	P-coumaroyl-quinic acid
							*m/z* 177.0546 [M + H-C_9_H_6_O_3_]^+^	
							*m/z* 145.0285 [M + H-C_9_H_6_O_5_]^+^	
13	8.76	[M + H]^+^	C_16_H_18_O_9_	355.0983	355.0989	4.5	*m/z* 355.0989 [M + H]^+^	Chlorogenic acid *
							*m/z* 193.0861 [M + H-C_9_H_6_O_3_]^+^	
							*m/z* 163.0837 [M + H-C_7_H_12_O_6_]^+^	
							*m/z* 145.0278 [M + H-C_7_H_14_O_7_]^+^	
							*m/z* 118.0864 [M + H-C_8_H_14_O_7_]^+^	
14	8.88	[M + Na]^+^	C_17_H_24_O_9_	395.1268	395.1269	0.9	*m/z* 395.1310 [M + Na]^+^	Eleutheroside B *
							*m/z* 373.1486 [M + H]^+^	
							*m/z* 211.0956 [M + H-Glu]^+^	
							*m/z* 193.0859 [M + H-Glu-H_2_O]^+^	
							*m/z* 161.0593 [M + H-Glu-H_2_O-CH_4_O]^+^	
15	8.95	[M + H]^+^	C_16_H_18_O_9_	355.0983	355.0979	3.2	*m/z* 355.0979 [M + H]^+^	Cryptochlorogenic acid
							*m/z* 163.0372 [M + H-C_7_H_12_O_6_]^+^	
16	9.03	[M + H]^+^	C_15_H_10_O_7_	303.2361	303.1340	4.7	*m/z* 303.1340 [M + H]^+^	Quercetin
17	9.42	[M + H]^+^	C_17_H_22_O_11_	403.1457	403.1448	3.7	*m/z* 403.1448 [M + H]^+^	Isofraxidin + Glu + H_2_O
							*m/z* 241.1474 [M + H-Glu]^+^	
							*m/z* 223.1446 [M + H-Glu-H_2_O]^+^	
18	9.58	[M + H]^+^	C_9_H_8_O_4_	181.0510	181.0504	4.4	*m/z* 181.0504 [M + H]^+^	Caffeic acid
							*m/z* 163.0398 [M + H-H_2_O]^+^	
19	9.93	[M + H]^+^	C_15_H_18_O_9_	343.0980	343.0991	3.5	*m/z* 377.1464 [M + H]^+^	Loganic acid
							*m/z* 342.1713 [M + H-H_2_O-OH]^+^	
20	10.08	[M + H]^+^	C_26_H_42_O_9_	499.1245	499.1259	3.7	*m/z* 499.1259 [M + H]^+^	Sumogaside
21	10.50	[M + H]^+^	C_8_H_16_O_6_	209.0054	209.0048	4.3	*m/z* 209.0048 [M + H]^+^	Eleutheroside C
22	10.60	[M + H]^+^	C17H20O9	369.1152	369.1153	1.3	*m/z* 369.1153 [M + H]^+^	3-Feruloylquinic acid
							*m/z* 177.0527 [M + H-C_7_H_11_O_6_]^+^	
							*m/z* 149.0572 [M + H-C_8_H_11_O_7_]^+^	
							*m/z* 17.0322 [M + H-C_9_H_14_O_8_]^+^	
23	10.81	[M + H]^+^	C_22_H_26_O_8_	419.1667	419.1669	1.5	*m/z* 419.1669 [M + H]^+^	Syringaresinol
							*m/z* 401.1564 [M + H-H_2_O]^+^	
							*m/z* 369.1294 [M + H-CH_6_O_2_]^+^	
24	10.81	[M + Na]^+^	C_34_H_46_O_18_	743.2711	743.2709	0.9	*m/z* 765.2535 [M + Na]^+^	Eleutheroside E *
							*m/z* 743.2750 [M + H]^+^	
							*m/z* 419.1720 [M + H-2Glu]^+^	
							*m/z* 401.1605 [M + H-2Glu-H_2_O]^+^	
25	11.12	[M + H]^+^	C_16_H_22_O_7_	327.1586	327.1595	3.8	*m/z* 327.1595 [M + H]^+^	Eugenol glucoside
							*m/z* 165.1624 [M + H-Glu]^+^	
26	11.72	[M + H]^+^	C_14_H_18_O_9_	331.1549	331.1514	4.5	*m/z* 331.1514 [M + H]^+^	1-*O*-Vanilloyl-beta-D-glucose
							*m/z* 313.1408 [M + H-H_2_O]^+^	
27	12.05	[M + H]+	C_14_H_16_O_4_	249.1130	249.1121	3.5	*m/z* 249.1121 [M + H]^+^	Prenyl caffeate
							*m/z* 231.1011 [M + H-H_2_O]^+^	
28	12.32	[M + H]^+^	C_11_H_10_O_5_	223.0585	223.0581	1.6	*m/z* 223.0615 [M + H]^+^	Isofraxidin *
							*m/z* 207.0291 [M + H-CH_3_]^+^	
							*m/z* 190.0265 [M + H-CH_3_-OH]^+^	
							*m/z* 162.0371 [M + H-2OCH_3_]^+^	
							*m/z* 134.0365 [M + H-2OCH_3_-CO]^+^	
29	13.11	[M + H]+	C_19_H_18_O_11_	423.2123	423.2031	2.6	*m/z* 423.0231 [M + H]^+^	Mangiferin
							*m/z* 405.1931 [M + H-H_2_O]^+^	
30	13.45	[M + H]^+^	C_20_H_22_O_6_	359.1489	359.1488	0.3	*m/z* 359.1488 [M + H]^+^	Pinoresinol
							*m/z* 171.1020 [M + H-C_9_H_16_O_4_]^+^	
31	13.84	[M + H]^+^	C_22_H_26_O_9_	435.1662	435.1613	3.7	*m/z* 435.1613 [M + H]^+^	Ciwujiatone
							*m/z* 417.1512 [M + H-H_2_O]^+^	
32	14.33	[M + H]^+^	C_20_H_24_O_6_	361.1687	361.1656	3.5	*m/z* 361.1656 [M + H]^+^	Lariciresinol
33	14.50	[M + H]^+^	C_15_H_10_O_6_	287.1238	287.1250	3.7	*m/z* 287.1250 [M + H]^+^	Kaempferol
							*m/z* 153.1250 [M + H-C_8_H_6_O_2_]^+^	
34	17.17	[M + H]^+^	C_20_H_22_O_6_	359.1420	359.1460	1.2	*m/z* 359.1460 [M + H]^+^	Matairesinol
							*m/z* 341.1354 [M + H-H_2_O]^+^	
							*m/z* 323.1245 [M + H-H_4_O_2_]^+^	
							*m/z* 137.0579 [M + H-C_12_H_13_O_4_]^+^	
35	18.75	[M + H]^+^	C_29_H_50_O	415.1523	415.1405	4.8	*m/z* 415.1405 [M + H]+	β-Sitosterol
36	19.19	[M + H]^+^	C_17_H_20_O_10_	385.1217	385.1287	4.2	*m/z* 385.1287 [M + H]^+^	Isofraxidin 7-*O*-beta-D-Glucoside
							*m/z* 223.1276 [M + H-Glu]^+^	
37	20.20	[M + H]^+^	C_20_H_20_O_6_	357.1453	357.1333	3.9	*m/z* 357.1333 [M + H]^+^	Balanophonin
							*m/z* 339.1202 [M + H-H_2_O]^+^	
38	21.76	[M + H]^+^	C_21_H_20_O_10_	433.2262	433.2236	3.8	*m/z* 433.2236 [M + H]^+^	Schizandrin
							*m/z* 415.2137 [M + H-H_2_O]^+^	
							*m/z* 384.1947 [M + H-H_2_O-C_2_H_5_]^+^	
							*m/z* 346.1423 [M + H-H_2_O-C_5_H_9_]^+^	
39	22.33	[M + H]^+^	C_35_H_60_O_6_	577.2864	577.2847	4.2	*m/z* 577.2847 [M + H]^+^	Eleutheroside A
							*m/z* 385.2795 [M + H-C_7_H_12_O_6_]^+^	
							*m/z* 191.2855 [M + H-C_28_H_50_]^+^	
40	23.16	[M + H]^+^	C_20_H_16_O_6_	353.0997	353.0991	2.5	*m/z* 353.0991 [M + H]^+^	Savinin
							*m/z* 335.0887 [M + H-H_2_O]^+^	
41	23.35	[M + H]^+^	C20H18O6	355.1101	355.1149	2.8	*m/z* 355.1149 [M + H]^+^	Sesamin
							*m/z* 337.1046 [M + H-H_2_O]^+^	
							*m/z* 135.0425 [M + H-C_12_H_12_O_4_]^+^	
42	23.53	[M + H]^+^	C_9_H_8_O_3_	165.0587	165.0547	3.7	*m/z* 165.0547 [M + H]^+^	4-Hydroxycinnamic acid
							*m/z* 147.0587 [M + H-H_2_O]^+^	
43	23.63	[M + H]^+^	C_25_H_24_O_12_	517.2246	517.2338	2.8	*m/z* 517.2338 [M + H]^+^	Isochlorogenic acid A
							*m/z* 499.2326 [M + H-H_2_O]^+^	
							*m/z* 193.2348 [M + H-C_18_H_12_O_6_]^+^	
							*m/z* 163.2287 [M + H-C_16_H_18_O_9_]^+^	
44	24.03	[M + H]^+^	C_10_H_10_O_4_	195.0651	195.0636	2.6	*m/z* 195.0636 [M + H]^+^	Ferulic acid
							*m/z* 149.0236 [M + H-COOH]^+^	
45	26.07	[M + H]^+^	C_31_H_45_NO_4_	496.3418	496.3418	0.7	*m/z* 496.3418 [M + H]^+^	Citreoviridin A
							*m/z* 184.0744 [M + H-C_20_H_26_NO_2_]^+^	
46	26.50	[M + H]^+^	C_17_H_22_O_10_	387.1832	387.1816	4.3	*m/z* 387.1816 [M + H]^+^	trans-sinapoyl β-D-glucoside
							*m/z* 225.1786 [M + H-Glu]^+^	

“*” represents the use of the standard solution for comparison.

#### 3.2.1 Identification of organic acids

**FIGURE 2:**
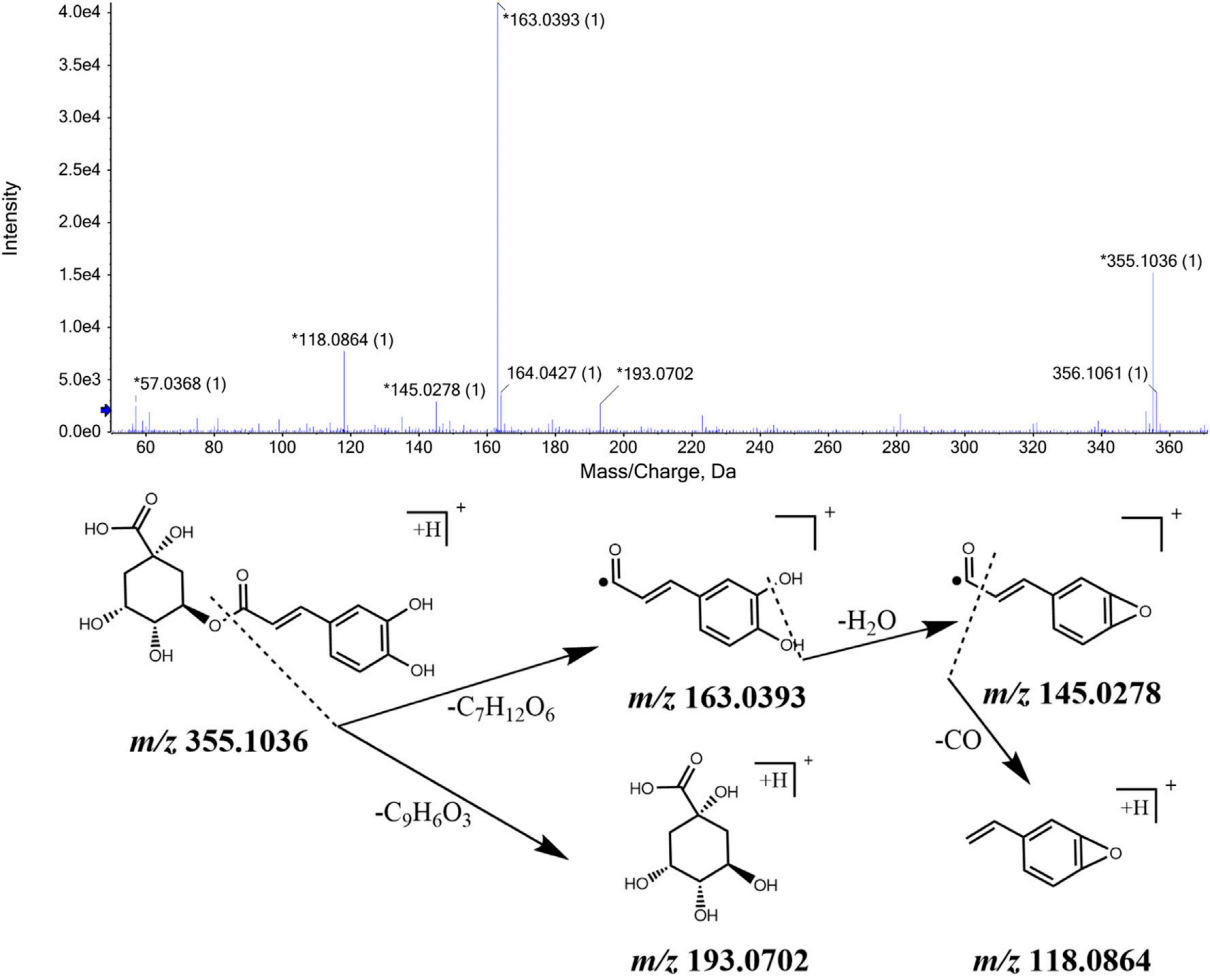
MS/MS spectrum of chlorogenic acid in CWT and speculated fragmentation pathway.

Based on MS/MS data and information obtained from the ETCM and TCMID databases, we identified 5-*O*-caffeoylshikimic acid, caffeic acid, ferulic acid, chlorogenic acid, cryptochlorogenic acid, phenylalanine, and other organic acid compounds in the CWT sample solution. They all have a carboxyl group and similar chemical structures. Their identification process takes chlorogenic acid as an example. The ion with *m/z* 355.1036 [M + H]^+^ was detected when the retention time was 8.76 min, and, through the comprehensive score, it was inferred that the molecular formula was C_16_H_18_O_9_. This molecular formula was consistent with that of chlorogenic acid in *Acanthopanax senticosus*. Besides, fragment ions with *m/z* 193.0702, m/z 163.0393, m*/z* 145.0278, and *m/z* 118.0864 were also detected. They were initially identified as chlorogenic acid according to relevant literature. It is speculated that the ion fragment of *m/z* 163.0393 could be obtained by removing a quinic acid ion from chlorogenic acid, and the ion with *m/z* 145.0278 was obtained by removing one H_2_O from that with *m/z* 163.0393. Furthermore, the *m/z* 145.0278 lost a carbonyl group and formed an *m/z* 118.0864 fragment. The MS/MS spectrum and the speculated cleavage pathway are shown in Figure 2. Further, the total ion current chromatogram of the chlorogenic acid reference standard solution was collected under the same measurement conditions, and its MS/MS spectrum is shown in Supplementary Figure S2. It was found that the retention time and MS/MS data were consistent with the corresponding peaks in the chromatogram of CWT. Therefore, it was identified as chlorogenic acid.

#### 3.2.2 Identification of phenylpropanoids

Phenylpropanoids are a class of natural compounds consisting of a benzene ring and three straight-chain carbon linkages (C6-C3 group). Generally, it has the structure of phenol. Based on MS/MS data and referring to ETCM and TCMID databases, we identified a large number of phenylpropanoid compounds in CWT sample solution, e.g., eleutheroside B, ciwujiatone, eleutheroside E, trans sinapyl β-D-glucoside, 3-ferroloylquinic acid, 4-hydroxycinmic acid, caffeic acid, ferrolic acid, pinoresinol, sesamin, syringaresinol, laricariesinol, etc. Among them, some chemical structures contain one C6-C3 group, and some are formed by the polymerization and oxidation of two C6-C3 groups.

**FIGURE 3:**
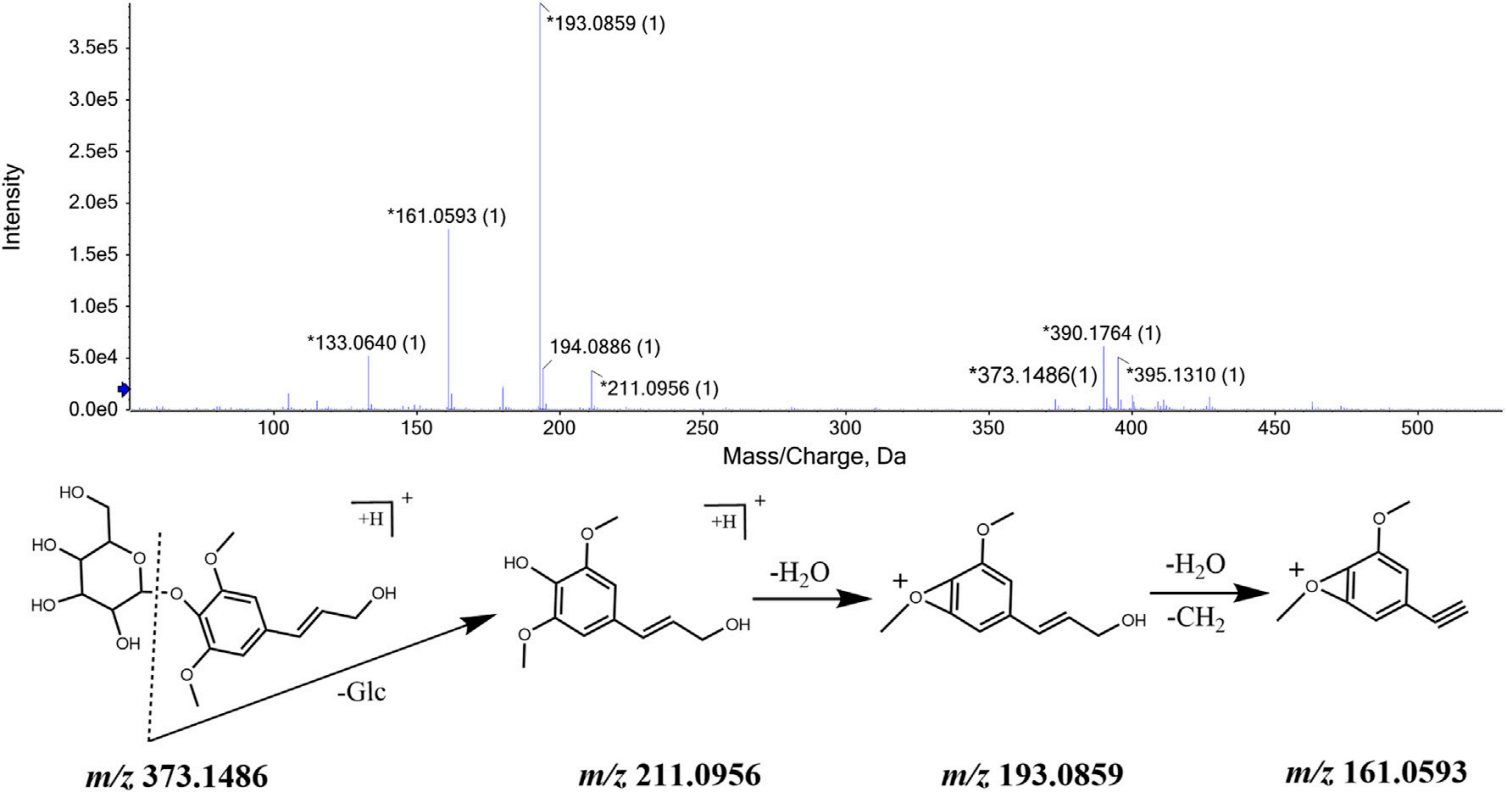
MS/MS spectrum of eleutheroside B in CWT and speculated fragmentation pathway.

Based on the number of C6-C3 groups in the chemical structure, we took eleutheroside B and eleutheroside E as examples and showed their identification process. The ions with *m/z* 373.1486 [M + H]^+^ and *m/z* 395.1310 [M + Na]^+^ were detected when the retention time was 8.88 min, and the comprehensive score pointed out that the molecular formula was C_17_H_24_O_9_. This molecular formula was consistent with that of eleutheroside B in *Acanthopanax senticosus*. Besides, fragment ions with *m/z* 211.0956, *m/z* 193.0859, and *m/z* 161.0593 were also detected. They were initially identified as eleutheroside B according to relevant literature. In addition, we speculated that *m/z* 211.0956 was obtained by removing a glucose molecule from the quasi-molecular ion peak *m/z* 373.1486, and the ion with *m/z* 193.0859 was obtained by removing one H_2_O from that with *m/z* 211.0956. The MS/MS spectrum and the speculated cleavage pathway are illustrated in Figure 3. Further, the total ion chromatogram of the eleutheroside B reference standard solution was collected under the same measurement conditions, and its MS/MS spectrum is shown in Supplementary Figure S3. We concluded that the retention time and MS/MS data were consistent with the corresponding peaks in the chromatogram of CWT. Therefore, it was identified as eleutheroside B.

**FIGURE 4:**
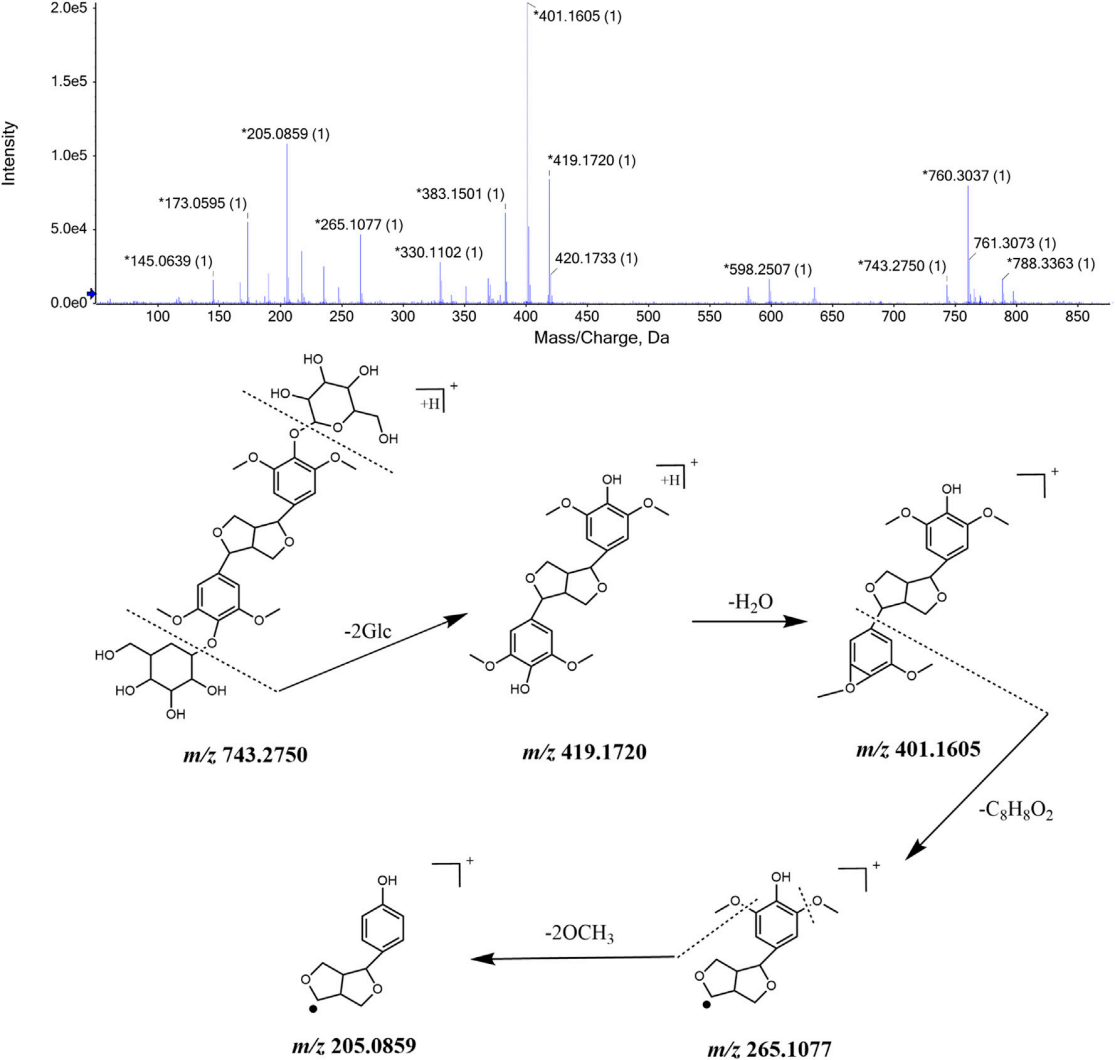
MS/MS spectrum of eleutheroside E in CWT and speculated fragmentation pathway.

Pinoresinol,ciwujiatone, eleutheroside E, sesamin, syringaresinol, lariciresinol are natural compounds formed by the oxidative polymerization of phenylpropanoid. All of these substances have similar chemical structure composition and fragmentation process and their identification process takes eleutheroside E as a basis. The ions with *m/z* 743.2750 [M + H]^+^ and *m/z* 765.2535 [M + Na]^+^ were detected when the retention time was 10.81 min, and through the comprehensive score, it was inferred that the molecular formula was C_34_H_46_O_18_. This molecular formula was consistent with that of eleutheroside E in *Acanthopanax senticosus*. Besides, fragment ions with m/z 419.1720, m/z 401.1613, and m/z 265.1077, m/z 205.0859 were also detected. It was speculated that *m/z* 419.1719 was produced by the loss of two molecules of glucose in *m/z* 743.2766, and *m/z* 401.1605 was obtained by removing one H_2_O from *m/z* 419.1720. The MS/MS spectrum and the speculated cleavage pathway are shown in Figure 4. Further, the total ion current chromatogram of the eleutheroside E reference standard solution was collected under the same measurement conditions, and its MS/MS spectrum is shown in Supplementary Figure S4. We concluded that the retention time and primary and MS/MS data were consistent with the corresponding peaks in the chromatogram of CWT. Therefore, it was identified as eleutheroside E.

#### 3.2.3 Identification of coumarins

**FIGURE 5:**
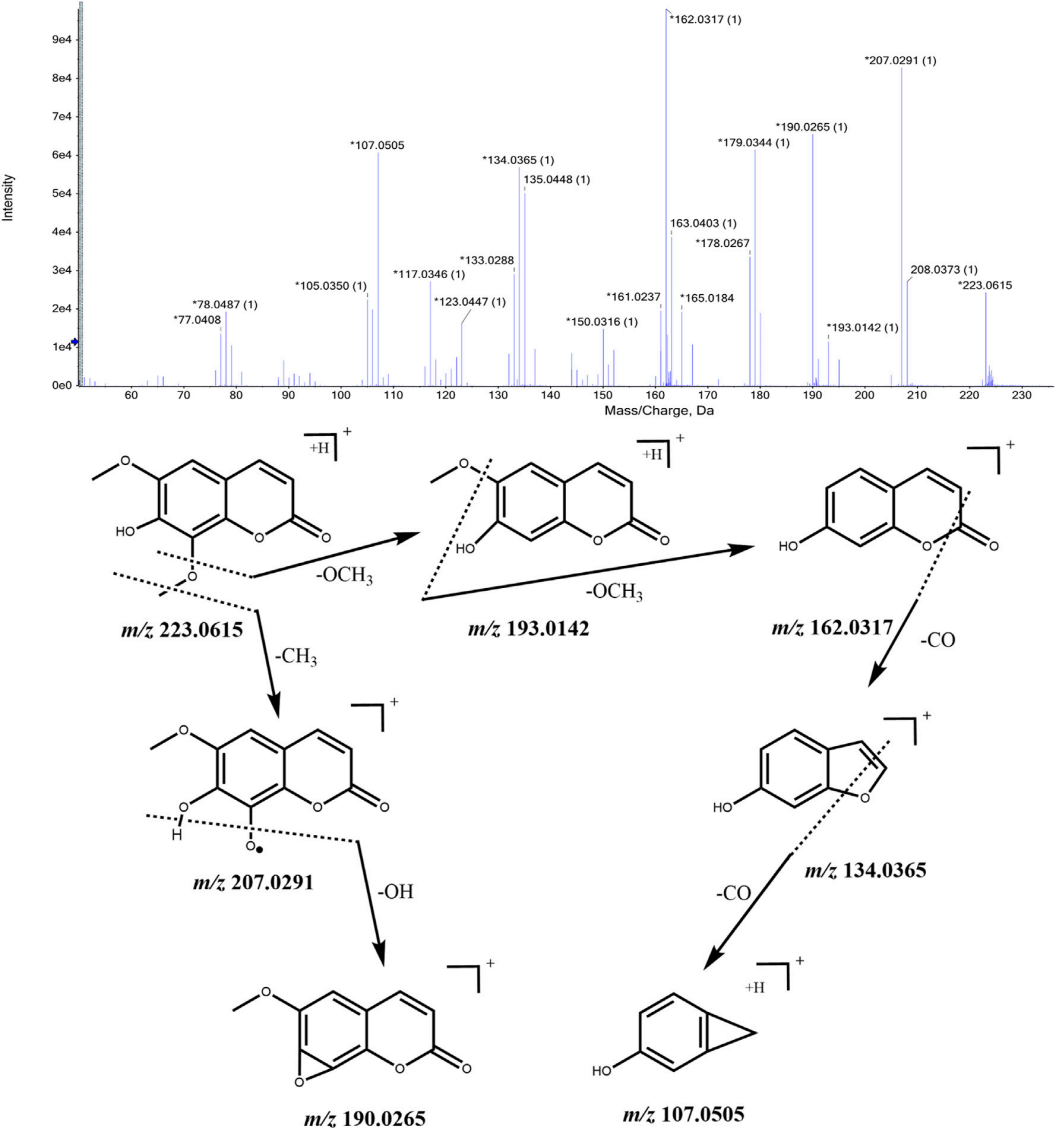
MS/MS spectrum of isofraxidin in CWT and speculated fragmentation pathway.

Coumarins are a class of lactone compounds formed by the dehydration and cyclization of 2-hydroxycinnamic acid. In plants, coumarins are often found in a free state or combined with sugars to form glycosides. Clausarin, isofraxidin, isofraxidin, and 7-*O*-beta-D-glucoside were identified in CWT sample solution. Their identification process takes isofraxidin as an example. The ion with *m/z* 223.0615 [M + H]^+^ was detected when the retention time was 12.32 min, and considering the comprehensive score, we inferred that the molecular formular was C_11_H_10_O_5_. This molecular formula was consistent with that of isofraxidin in *Acanthopanax senticosus*. Besides, fragment ions with *m/z* 207.0291, *m/z* 193.0142, m/z 190.0265, m/z 162.0317, m/z 134.0365, and *m/z* 107.0505 were also detected. It was speculated from the mass spectrum that *m/z* 207.0291 was obtained by removing one -CH_3_ from *m/z* 223.0615, and *m/z* 193.0142 was obtained by removing one -OCH_3_ from *m/z* 223.0615. In addition, *m/z* 193.0142 lost one -OCH_3_ formed an *m/z* 162.0317 fragment. Furthermore, the *m/z* 162.0317 lost a carbonyl group and formed an *m/z* 134.0365 fragment. They were initially identified as isofraxidin according to relevant literature (Huang et al., 2020). The MS/MS spectrum and the speculated cleavage pathway are shown in Figure 5. Further, the total ion current chromatogram of the isofraxidin reference standard solution was collected under the same measurement conditions, and its MS/MS spectrum is shown in Supplementary Figure S5. We concluded that the retention time and MS/MS data were consistent with the corresponding peaks in the chromatogram of CWT. Therefore, it was identified as isofraxidin.

### 3.3 Exploration of the mechanism of ciwujia tablet in the treatment ofinsomnia by network pharmacology

#### 3.3.1 Screening of ciwujia tabletingredients

As an effective drug, potent molecules must reach the target in the body in sufficient concentration and stay it for a long time in the form of biological activity. The Brain Or IntestinaL EstimateD permeation method (BOILED-Egg) is proposed as an accurate predictive model that works by computing the lipophilicity and polarity of small molecules. The BOILED-Egg method can be applied in a variety of settings, from the filtering of chemical libraries at the early steps of drug discovery to the evaluation of drug candidates for development. The SwissADME web tool gives free access to a pool of fast yet robust predictive models for physicochemical properties, pharmacokinetics, drug-likeness and medicinal chemistry friendliness, among which in-house proficient methods such as the BOILED-Egg, and bioavailability radar. The SwissADME web tool is a new method that integrates many other techniques used to screen promising compounds. It has advantageous features such as high efficiency and good accuracy. Here, we used the SwissADME web tool to screen all the CWT ingredients. The solubility class, GI absorption, and bioavailability score were selected as screening indicators. Then, those ingredients with good solubility, favorable gastrointestinal absorption, and high bioavailability were selected for subsequent experiments. A total of 17 ingredients were selected according to the above conditions, as listed in Table 2.

**TABLE 2:** CWT active ingredients screening results.

No	Ingredient name	Molecular weight	Solubility class	GI absorption	Bioavailability score
1	Tyrosine	181.19 g/mol	Highly soluble	High	0.55
2	Liriodenine	275.26 g/mol	Moderately soluble	High	0.55
3	Phenylalanine	165.19 g/mol	Highly soluble	High	0.55
4	Quercetin	302.24 g/mol	Soluble	High	0.55
5	Caffeic acid	180.16 g/mol	Soluble	High	0.56
6	Eleutheroside C	208.21 g/mol	Highly soluble	High	0.55
7	Syringaresinol	418.44 g/mol	Soluble	High	0.55
8	Eugenol glucoside	326.34 g/mol	Very soluble	High	0.55
9	Isofraxidin	222.19 g/mol	Soluble	High	0.55
10	Ciwujiatone	434.44 g/mol	Soluble	High	0.55
11	Lariciresinol	360.40 g/mol	Soluble	High	0.55
12	Kaempferol	286.24 g/mol	Soluble	High	0.55
13	Matairesinol	358.39 g/mol	Moderately soluble	High	0.55
14	Savinin	352.34 g/mol	Moderately soluble	High	0.55
15	Sesamin	354.35 g/mol	Soluble	High	0.55
16	trans-p-coumaric acid	164.16 g/mol	Soluble	High	0.85
17	Ferulic acid	194.18 g/mol	Soluble	High	0.85

#### 3.3.2 Construction of the ingredient-target network diagram

The SwissTargetPrediction web tool (Gfeller et al., 2013) (http://www.swisstargetprediction.ch/) was adopted to predict the targets of 17 ingredients. The targets with a probability score larger than zero were selected through probability score screening. A total of 377 targets including EGFR, CA9, ADORA2A, CA12, GSK3B were collected, and their target network was constructed by Cytoscape 3.8.0 software (refer to Supplementary Figure S6).

#### 3.3.3 Ingredient-disease commonpotential targets

The insomnia-related targets related to insomnia were screened based on data obtained from the Genecards (https://www.genecards.org/) and OMIM (https://omim.org/) databases, and a total of 2,934 targets including synuclein alpha, interleukin 6, delayed sleep phase syndrome, proline dehydrogenase 1, and restless legs syndrome were collected (Supplementary Table S2). These 17 ingredient targets and disease targets were mapped through the Venny platform, and 134 crossover targets including angiotensin I converting enzyme, acetylcholinesterase, adrenoceptor alpha 1A, dopamine receptor D1, D2, D3, D4, D5, gamma-aminobutyric acid type A receptor subunit 2, alpha1, beta3, gamma2, glutamate metabotropic receptor 4, 5-hydroxytryptamine receptor 1A, 1B, 2A, 2C were obtained (Supplementary Figure S7; Supplementary Table S3). We concluded that these 17 ingredients may exert therapeutic effects on insomnia through the 134 crossover targets.

#### 3.3.4 Construction of the protein-protein interaction network diagram ofkey anti-insomnia targets

The STRING (a search tool used to retrieve interacting genes) database (https://string-db.org/) was included in our methodology. Homo sapiens species were selected to draw the protein-protein interaction (PPI) network of 134 common targets, and the key targets were found through topological analysis. The results showed that MTNR1A and MTNR1B were free targets, which means that they did not intersect with other targets. The PPI network was obtained after hiding these two free targets. There were 132 nodes and 1195 edges in the network, as shown in Supplementary Figure S8.

**FIGURE 6:**
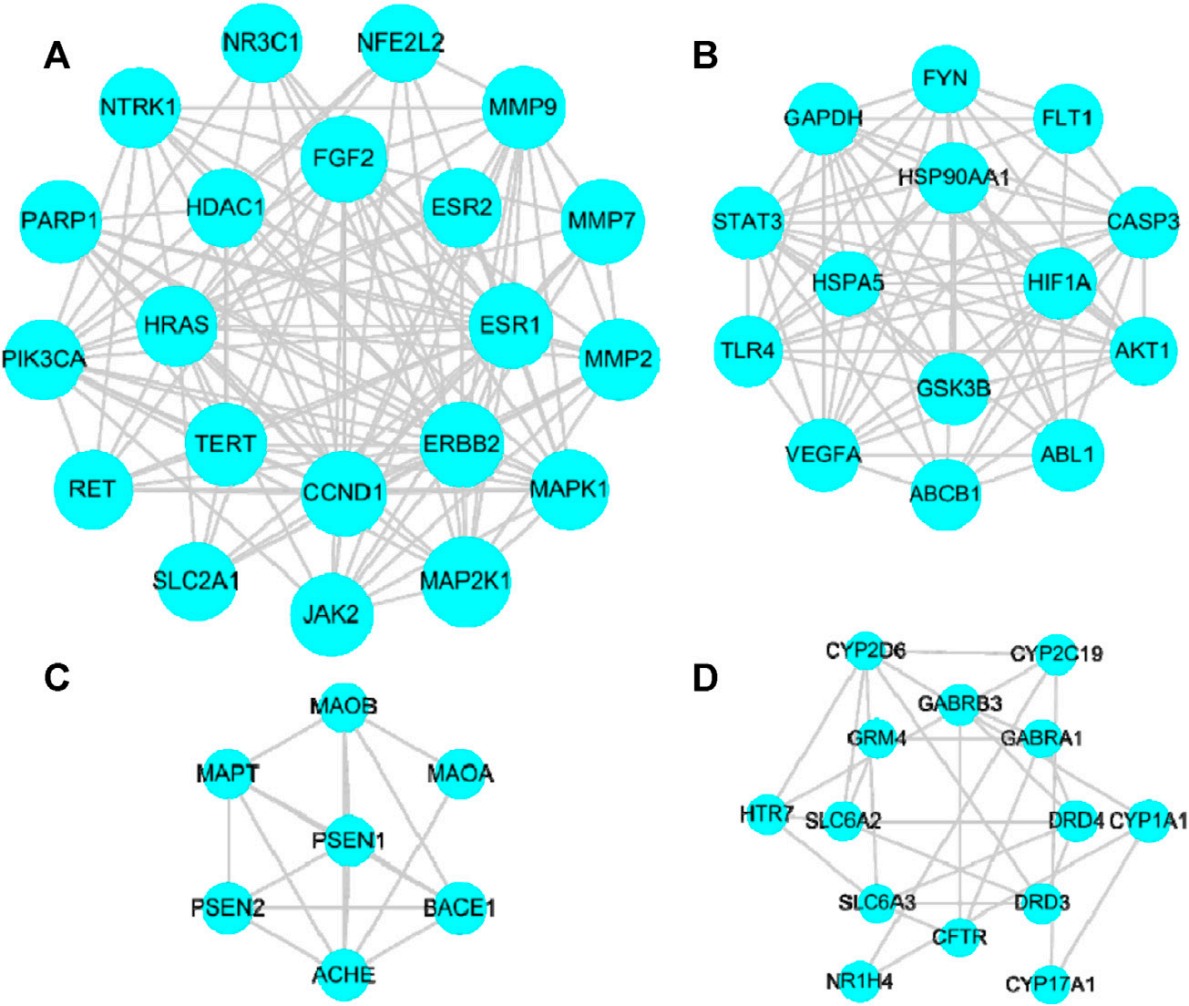
Significant clusters analysis. **(A)**: Cluster module (score = 12.30), including 21 nodes and 123 edges; **(B)**: Cluster module (score = 11.38), including 14 nodes and 74 edges; **(C)**: Cluster module (score = 5.33), including 7 nodes and 16 edges; **(D)**: Cluster module (score = 4.00), including 14 nodes and 26 edges.

We used the molecular complex detection (MCODE) plugin in Cytoscape 3.8.0 to perform the cluster analysis of PPI networks, and the parameters were set as degree cutoff = 5, node score cutoff = 0.2, k-core = 2, and max. depth = 100. During the procedure, four cluster modules were yielded, as shown in Figure 6. Larger nodes in the figure are associated with greater degree values. These high-degree protein targets may be the key targets such as neurotrophic receptor tyrosine kinase 1, nuclear receptor subfamily 3 group C member 1, cyclin D1, erb-B2 receptor tyrosine kinase 2, and account for the essential therapeutic effects of CWT on insomnia. However, as the number of nodes in this PPI network was moderate and the network was not too complex, GO and KEGG analysis could be performed on all 132 targets in the PPI network to avoid omitting the analysis of any node. This effort can ensure the integrity and accuracy of the CWT’s mechanism in treating insomnia.

#### 3.3.5 Gene ontology and KEGG pathway enrichment analysis

**FIGURE 7:**
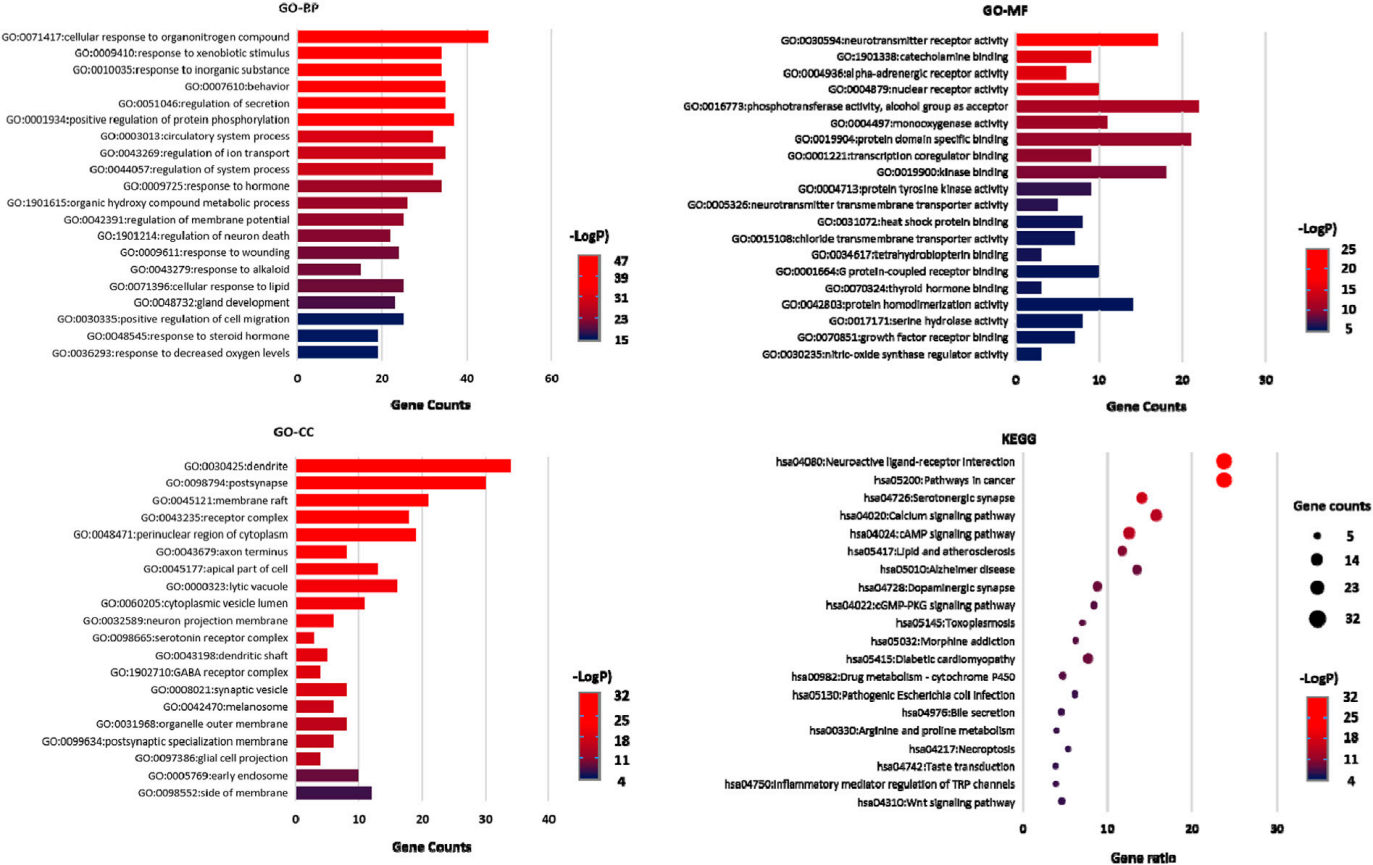
GO-BP, GO-MF, and GO-CC enrichment analysis diagram and KEGG pathway enrichment analysis diagram.

KEGG pathway enrichment and Gene Ontology (GO) enrichment analyses were performed on 132 key targets through the Metascape data platform (https://metascape.org/). Homo sapiens was selected as the analysis species, and the *p*-value was < 0.01. GO enrichment includes GO Biological Processes (GO-BP), GO Molecular Functions (GO-MF), and GO Cellular ingredients (GO-CC). A total of 1813 entries were obtained through GO enrichment analysis, including 1515 in GO-BP, 130 in GO-CC, and 168 in GO-MF. The top 20 analysis results were selected according to the -Log *p* value to draw the GO functional enrichment map. A total of 167 entries were obtained from the KEGG pathway enrichment analysis. The top 20 pathways were listed and plotted according to the *p*-value, as shown in Figure 7.

Through GO functional enrichment analysis, we identified that the enrichment results were mainly related to the regulation of neurotransmitter levels, neurotransmitter metabolic process, modulation of ion transport, behavior, monoamine transport, negative regulation of synaptic transmission, synaptic transmission, dopaminergic transmission, synapse organization and other processes. The enrichment analysis results of the KEGG pathway mainly involved neuroactive ligand-receptor interaction, serotonergic synapse, dopaminergic synapse, cAMP signaling pathways, calcium signaling pathways, Alzheimer’s disease, lipid and atherosclerosis, prolactin signaling pathways, Gap junction, morphine addiction, Parkinson’s disease and other pathways. Based on these analysis results, we concluded that these CWT ingredients were closely related to the metabolism, transport, and activity regulation of neurotransmitters in the brain. These biological processes were also closely related to the treatment of insomnia with CWT.

### 3.4 Animal experiment verification

#### 3.4.1 Effects on sleep latency

**FIGURE 8:**
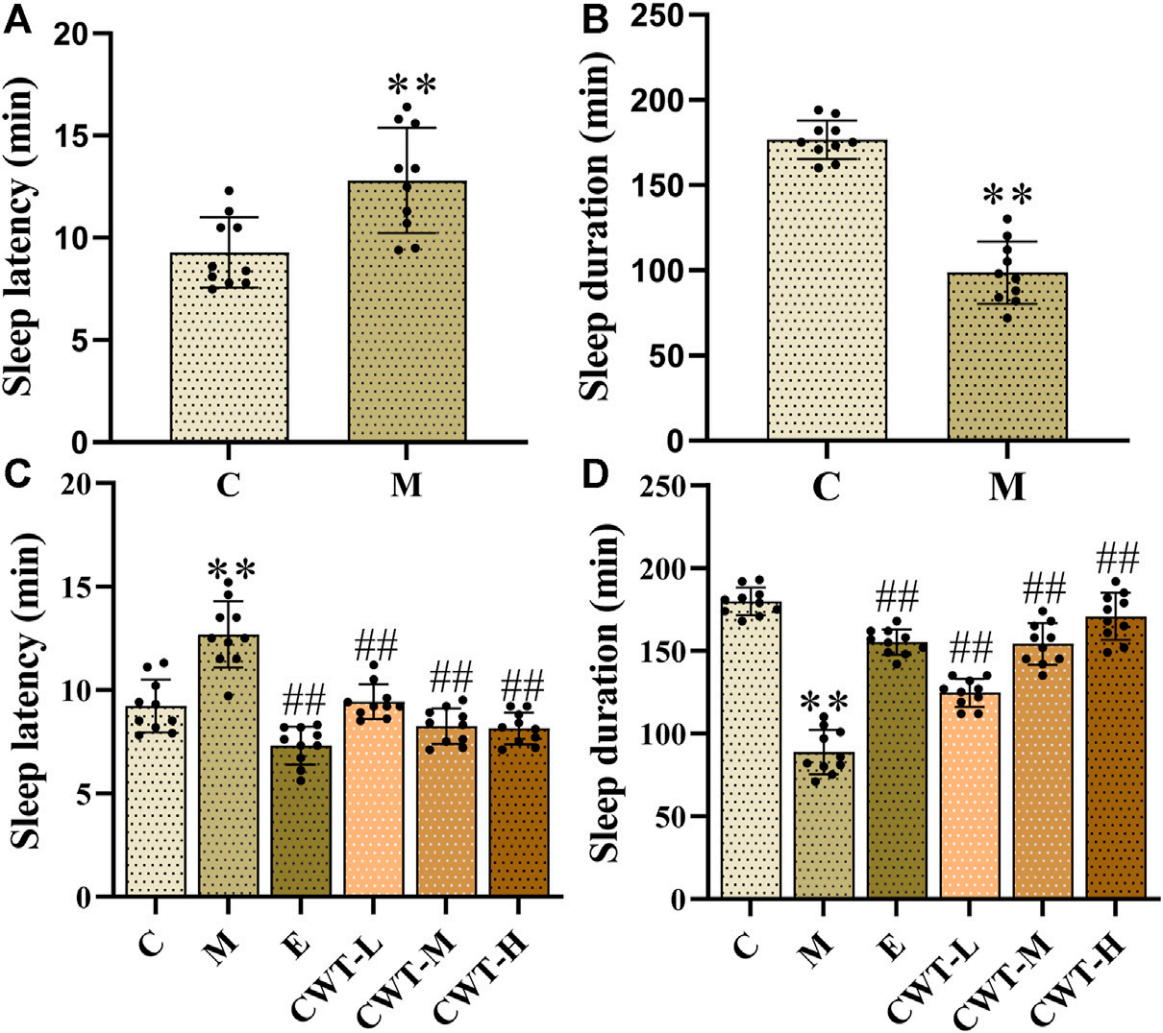
Sleep test results (C: control group, *n* = 10; M: model group, *n* = 10; E: estazolam group, *n* = 10; CWT-L group, *n* = 10; CWT-M group, *n* = 10; CWT-H group, *n* = 10; compared with the group C, *p < 0.05, **p < 0.01; compared with the group M, #*p* < 0.05, ##*p* < 0.01, mean ± SD). Figures **(A,B)**: Test results on the fourth day of insomnia model establishment; Figures **(C,D)**: Test results on the seventh day of treatment. **(A)**: Detection of sleep latency of rats in groups C and M. **(B)**: Detection of sleep duration of rats in groups C and M. **(C)**: Detection of sleep latency of rats in each group. **(D)**: Detection of sleep duration of rats in each group.

After the insomnia model was established, we observed that, compared with group C, the sleep latency of rats in the M group was significantly prolonged (*p* < 0.05) (Figure 8A). After 7 days of treatment, we also observed that the sleep latency of rats in group M was significantly prolonged (*p* < 0.01) when comparing the results with group C. Compared with group M, the sleep latency of rats in the CWT treatment group and the estazolam (E) group decreased significantly (*p* < 0.01). Among them, the most significant decrease in sleep latency was found in group CWT-H, and the effect was better than that in group E (Figure 8C).

#### 3.4.2 Effects on sleep duration

After the establishment of the insomnia model, the sleep duration of rats in group M decreased significantly (*p* < 0.05) compared with group C (Figure 8B), demonstrating that the model was successfully designed. On the seventh day of treatment, the sleep duration of rats in group M decreased significantly compared with group C (*p* < 0.01). Compared with group M, the sleep duration of rats in the CWT treatment group and group E was significantly prolonged (*p* < 0.01). CWT achieved a favorable effect on prolonging the sleep duration of insomnia rats. Furthermore, the high-dose CWT had the most significant impact on prolonging the sleep duration of insomnia rats, and the results were similar to those of group E (Figure 8D).

#### 3.4.3 Effects on the neurotransmitter content in the brain

The GO and KEGG analysis results indicated that the obtained pathways closely correlated with neurotransmitters, such as neuroactive ligand-receptor interaction, serotonergic synapse, and dopaminergic synapse, ranked high in related results. Therefore, 10 rats were randomly selected from the groups C and M, respectively, on the fourth day of model establishment. The level of neurotransmitters in the rats’ brains was measured, including 5-HT, GABA, DA, and NE. The sample preparation and detection methods were conducted under ice bath conditions. First, 5.0 ml PBS was added per 1.0 g of brain tissue to prepare a uniform sample solution. The sample solution was then centrifuged at 3,000 rpm and 4 C for 20 min to obtain the supernatant. We strictly followed the guidelines on the ELISA kit for detection. All the rats in different groups were also tested on the seventh day of treatment.

**FIGURE 9:**
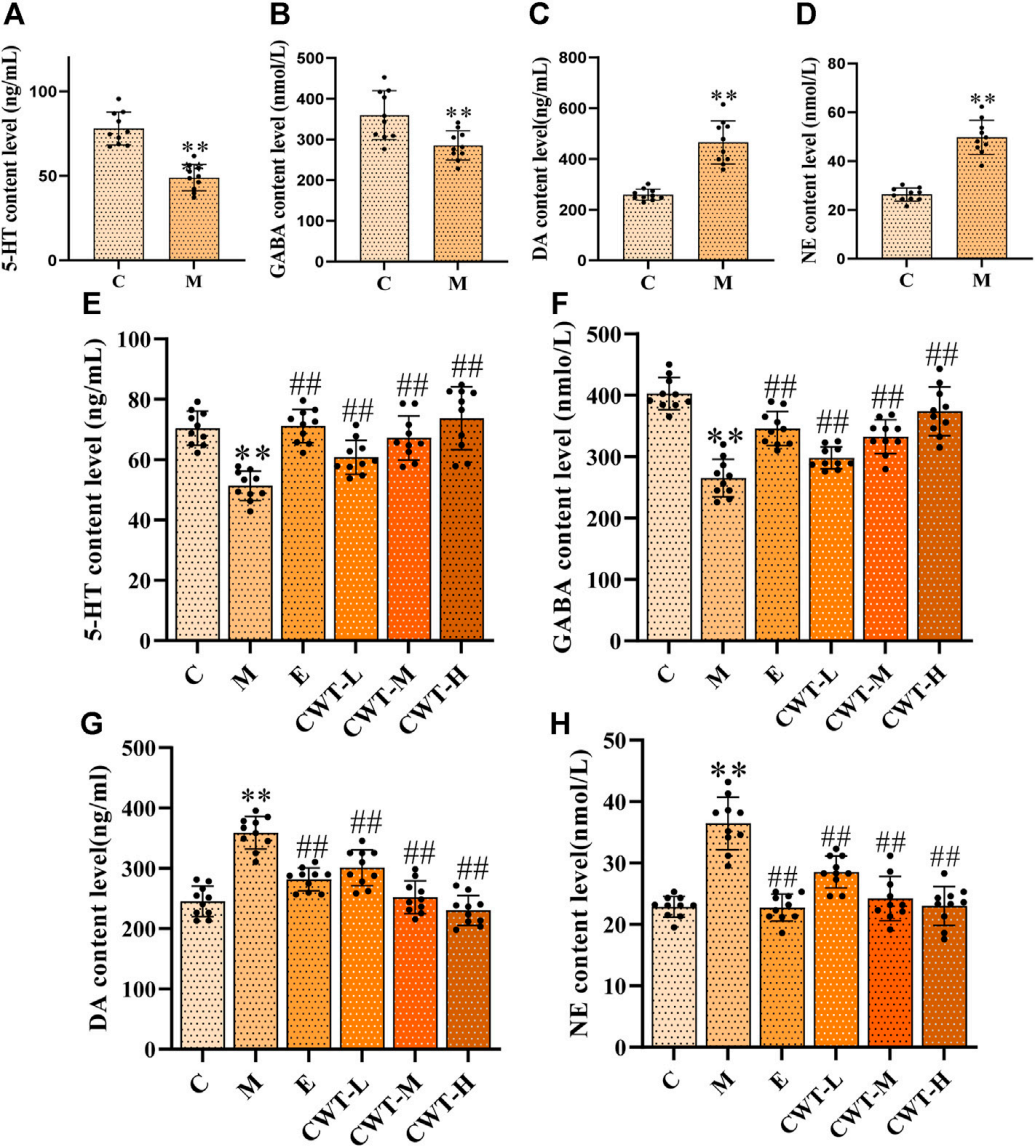
Neurotransmitter content in the brain (C: control group, n = 10; M: model group, *n* = 10; E: estazolam group, *n* = 10; CWT-L group, *n* = 10; CWT-M group, *n* = 10; CWT-H group, *n* = 10; compared with the group C, *p < 0.05, **p < 0.01; compared with the group M, #*p* < 0.05, ##*p* < 0.01, mean ± SD). Figures **(A–D)**: Test results on the fourth day of insomnia model establishment; Figures **(E–H)**: Test results on the seventh day of treatment. **(A)**: 5-HT content analysis in groups C and M. **(B)**: GABA content analysis in groups C and M. **(C)**: NE content analysis in groups C and M. **(D)**: DA content analysis in groups C and M. **(E)**: Analysis of the 5-HT content in the brain of rats in each group. **(F)**: Analysis of the GABA content in each group. **(G)**: Analysis of the DA content in each group. **(H)**: Analysis of the NE content in each group.

On the fourth day of insomnia model establishment, we identified that, compared with group C, the content of 5-HT and GABA in the brain of rats from group M decreased, while that of DA and NE increased significantly (*p* < 0.01) (Figures 9A–D). On the seventh day of treatment, the content of these four neurotransmitters in the brain of rats in each administration group was considerably different from those in group M (*p* < 0.05). Compared with group M, the content of 5-HT and GABA in the brain of rats in the CWT treatment group and group E increased significantly, while that of DA and NE decreased significantly. Thus, we concluded that the therapeutic effect of CWT was similar to that of estazolam (Figures 9E–H).

## 4 Discussion

Insomnia is the most common sleep disorder and has drawn the attention of scholars and medical professionals around the world. In recent years, it has been explored and studied by many researchers in the field, shedding light on important discoveries related to this condition. Some studies have already proved that there are many factors linked to insomnia, including daily life stress, depression, and long-term brain tension. Other scholars also found that insomnia is closely related to the metabolic function of brain tissues such as locus coeruleus, amygdala, and hippocampus (Van Someren, 2021; Zou et al., 2021). At present, benzodiazepine receptor agonists are conventional drugs used in the treatment of insomnia. Due to the safety limitations of these drugs, other alternative therapies have been advocated (Robertson et al., 2019). This study analyzes the potential pathways and targets of CWT in the treatment of insomnia. Our methodology, combined with animal experiments, showed that CWT can alleviate insomnia symptoms by improving and restoring the brain’s stable state of neurotransmitter levels.

Natural medicines and natural medicine-derived products have received increasing attention of researchers worldwide. Studies have pointed out the efficacy of herbs, herbal extracts, and different parts of plant natural products to treat various diseases since prehistoric times (Goni et al., 2021). The diversity and complexity of their ingredients endow these resources with many pharmacological features. An extensive exploration on natural medicines could reveal their exact pharmacological effects and their favorable mechanisms in the treatment of related diseases, which can help people amplify the use of natural medicines in daily life. CWT is a product derived from ES, a plant with antifatigue, anti-depression, and anxiolytic properties (Liu et al., 2022).

In our experiment, 46 ingredients in CWT were identified by the UPLC-Q-TOF-MS/MS technique. Among them, some unique ingredients such as eleutheroside B, eleutheroside E, isofraxidin, and ciwujiatone were recognized, and the potential cracking pathways were speculated based on MS/MS data. Furthermore, we reviewed previous studies on ES according to the analysis results of UPLC-Q-TOF-MS/MS and found that it has many pharmacological effects. Some of its components have been previously studied by scholars in the field and their outcomes are very relevant to the community. Research shows that syringin can exert anti-breast cancer effects through PI3K-AKT and EGFR-RAS-RAF pathways (Wang et al., 2022), and their study revealed that Acanthopanax senticosus polysaccharide (ASPS) plays a major role in regulating immune damage caused by radiation. They also confirmed that this substance can regulate the intestinal p38MAPK-SKN-1/ATF-7 pathway and alleviate the body’s stress response (Liu et al., 2022).

Currently, CWT is mostly used in treating insomnia in clinical practice. In order to clarify the mechanism of CWT as an effective substance to treat this condition, it is necessary to comprehensively explore its ingredients, which was also the goal of our experiment. The UPLC-Q-TOF-MS/MS technique can efficiently identify active components of natural medicines, and it can be applied to evaluate the similarities and differences of these substances in different regions, as well as their different features. The findings presented in this study are of great significance for the structural modification of active ingredients of CWT and the improvement of pharmacodynamic functions in subsequent studies.

During the network pharmacology analysis, the swissADME web tool was used as an active compound screening tool that combines different methods such as BOILED-Egg permeation, bioavailability radar, and iLOGP. It can be applied in a variety of settings, from the filtering of chemical libraries at the early steps of drug discovery to the evaluation of drug candidates for further development. The solubility class, GI absorption, and bioavailability scores were used as indicators to screen CWT ingredients and ensure the accuracy of the analysis results. Besides, relevant literature reviews were performed based on the network pharmacology analysis results of CWT’s mechanism in the treatment of insomnia. Furthermore, results from the GO and KEGG analyses suggest that the pathways regulated by the active components of CWT closely correlate with neurotransmitters. This result has also been confirmed by other scholars who studied the mechanisms and factors associated with insomnia (Blake et al., 2018; Cho et al., 2021). Some inhibitory neurotransmitters such as 5-HT and GABA (Kim et al., 2019; Bruni et al., 2021) and excitatory neurotransmitters DA and NE (Broese et al., 2012; Dauvilliers et al., 2015) play a vital role in regulating people’s daily behavior, sleep-wake habits, cognition, and emotions. Among them, 5-HT is particularly important and it can effectively regulate sleep-wake behavior (Vaseghi et al., 2021).

An insomnia rat model was established by intraperitoneal injection of p-chlorophenylalanine to verify the reliability of GO and KEGG analysis results. The content of 5-HT, GABA, DA, and NE in the brains of insomnia rats was measured before and after CWT treatment. It was concluded that CWT can increase the content of inhibitory neurotransmitters 5-HT and GABA in the brain and reduce the synthesis of excitatory escalating transmitters DA and NE. Moreover, after testing the sleep status of these rats, we concluded that CWT treatment can shorten their sleep latency and prolong their sleep time. The accuracy and reliability of the results from the GO functional enrichment and KEGG pathway enrichment analyses were verified through animal experiments.

In addition, the cAMP signaling pathways, calcium signaling, Gap junction and other related pathways in the GO and KEGG analysis results have also been verified in related reports. As per some studies, sleep and wakefulness are closely related to the expression level of cAMP (Choi et al., 2016; Carev and Sarikurkcu, 2021), and it has been previously proved that sleep- and wake-promoting neuropeptide signals could decrease and increase cAMP levels during sleep (Cianciulli et al., 2019). Furthermore, taking into consideration that calcium signaling pathways underlie the regulation of sleep duration in mammals (Tatsuki et al., 2016), damage to this specific pathway will lead to increased wakefulness and abnormal brain rhythms (Bojarskaite et al., 2020). The GAP junction function is critical for healthy sleep (Huang L. et al., 2018) and CWT can regulate these pathways, thus generating therapeutic effects on insomnia. Our findings can offer further guidance for studies regarding the clinical medication features and compatibility of CWT in the treatment of insomnia.

It is equally important to acknowledge the limitations of this study. Firstly, some ingredients may have been ignored in the process of component identification due to their complex structures. Secondly, the TCM chemical database is constantly being supplemented and improved, and there may be some unknown ingredients. Furthermore, regardless of the results presented in this study, further examinations are still necessary to clarify the dose-effect relationship between the efficacy and ingredients of CWT in the treatment of insomnia.

## 5 Conclusion

In this study, 46 chemical ingredients in CWT were identified by UPLC-Q-TOF-MS/MS technique. Among them, 17 ingredients, including eleutheroside C, ciwujiatone, liriodenine, syringaresinol, isofraxidin, kaempferol, caffeic acid, and quercetin could regulate neurotransmitter levels, neurotransmitter metabolic processes, neuroactive ligand-receptor interaction, serotonergic synapse, dopaminergic synapse, and cAMP signaling pathways. In particular, it was identified that CWT could regulate the levels of 5-HT, GABA, DA, and NE neurotransmitters in the brain, ensuring their steady state to achieve a satisfactory effect in the treatment of insomnia. The results of this study can provide a foundation for further clinical application and related studies and also offer a new direction for developing natural medicines.

## Data Availability

The original contributions presented in the study are included in the article/Supplementary Material, further inquiries can be directed to the corresponding author.
