# Supplementary material for: Exploring potential mechanism of ciwujia tablets for insomnia by UPLC-Q-TOF-MS/MS, network pharmacology, and experimental validation
Source: Front Pharmacol. 2022 Aug 30;13:990996. doi: 10.3389/fphar.2022.990996 (PMC9468710; doi:10.3389/fphar.2022.990996)
Supplement: Supplementary file 3 [file Table1.DOCX]

| **Supplementary Table 1.**  Ingredients identified in CWT based on UPLC-Q-TOF MS/MS. (Negative model) | | | | | | | | |
| --- | --- | --- | --- | --- | --- | --- | --- | --- |
| No. | Peak Appearance Time (min) | Ion Species | Molecular Formula | Theoretical  Value (m/z) | Measured  Value (m/z) | Deviation  ΔPPM | Ion Fragment (m/z) | Ingredient Name |
| 1 | 1.37 | [M-H]^-^ | C_24_H_28_O_4_ | 379.0844 | 379.0830 | 3.8 | m/z 379.0830 [M-H]^-^  m/z 364.0859 [M-H-CH_3_]^-^  m/z 168.0564 [M-H-CH_3-_C_10_H_12_O_4_]^-^ | Clausarin |
| 9 | 7.11 | [M-H]^-^ | C_26_H_26_O_12_ | 529.1756 | 529.1566 | 1.9 | m/z 529.1566 [M-H]^-^  m/z 515.1391 [M-H-CH_2_]^-^ | 1-caffeoyl-5-feruloylquinic acid |
| 10 | 7.60 | [M-H]^-^ | [C](https://pubchem.ncbi.nlm.nih.gov/#query=C16H18O9)_[16](https://pubchem.ncbi.nlm.nih.gov/#query=C16H18O9)_[H](https://pubchem.ncbi.nlm.nih.gov/#query=C16H18O9)_[18](https://pubchem.ncbi.nlm.nih.gov/#query=C16H18O9)_[O](https://pubchem.ncbi.nlm.nih.gov/#query=C16H18O9)_[9](https://pubchem.ncbi.nlm.nih.gov/#query=C16H18O9)_ | 353.0891 | 353.0878 | 3.8 | m/z 353.0878 [M+H]^+^  m/z 191.0564 [M-H-C_10_H_8_O_3_]^-^ | Neochlorogenic acid |
| 13 | 8.90 | [M-H]^-^ | [C](https://pubchem.ncbi.nlm.nih.gov/#query=C16H18O9)_[16](https://pubchem.ncbi.nlm.nih.gov/#query=C16H18O9)_[H](https://pubchem.ncbi.nlm.nih.gov/#query=C16H18O9)_[18](https://pubchem.ncbi.nlm.nih.gov/#query=C16H18O9)_[O](https://pubchem.ncbi.nlm.nih.gov/#query=C16H18O9)_[9](https://pubchem.ncbi.nlm.nih.gov/#query=C16H18O9)_ | 353.0983 | 355.0877 | 4.1 | m/z707.1850[2M-H]^-^  m/z353.0983[M-H]^-^  m/z191.0564[M-H-Glu]^-^ | Chlorogenic acid * |
| 15 | 9.12 | [M-H]^-^ | [C](https://pubchem.ncbi.nlm.nih.gov/#query=C16H18O9)_[16](https://pubchem.ncbi.nlm.nih.gov/#query=C16H18O9)_[H](https://pubchem.ncbi.nlm.nih.gov/#query=C16H18O9)_[18](https://pubchem.ncbi.nlm.nih.gov/#query=C16H18O9)_[O](https://pubchem.ncbi.nlm.nih.gov/#query=C16H18O9)_[9](https://pubchem.ncbi.nlm.nih.gov/#query=C16H18O9)_ | 353.0891 | 353.0885 | 1.8 | m/z707.1861[2M-H]^-^  m/z353.0885[M-H]^-^  m/z173.0466[M-H-Glu-H_2_O]^-^ | Cryptochlorogenic acid |
| 22 | 10.85 | [M-H]^-^ | [C](https://pubchem.ncbi.nlm.nih.gov/#query=C17H20O9)_[17](https://pubchem.ncbi.nlm.nih.gov/#query=C17H20O9)_[H](https://pubchem.ncbi.nlm.nih.gov/#query=C17H20O9)_[20](https://pubchem.ncbi.nlm.nih.gov/#query=C17H20O9)_[O](https://pubchem.ncbi.nlm.nih.gov/#query=C17H20O9)_[9](https://pubchem.ncbi.nlm.nih.gov/#query=C17H20O9)_ | 367.1021 | 367.1014 | 1.9 | m/z367.1014[M-H]^-^  m/z191.0559[M-H-C_10_H_8_O_3_]^-^  m/z173.0454[M-H-C_10_H_8_O_3_-H_2_O]^-^ | 3-Feruloylquinic acid |

“*” represents the use of the standard solution for comparison.
