# Supplementary material for: Exploring potential mechanism of ciwujia tablets for insomnia by UPLC-Q-TOF-MS/MS, network pharmacology, and experimental validation
Source: Front Pharmacol. 2022 Aug 30;13:990996. doi: 10.3389/fphar.2022.990996 (PMC9468710; doi:10.3389/fphar.2022.990996)
Supplement: Supplementary file 4 [file DataSheet1.docx]

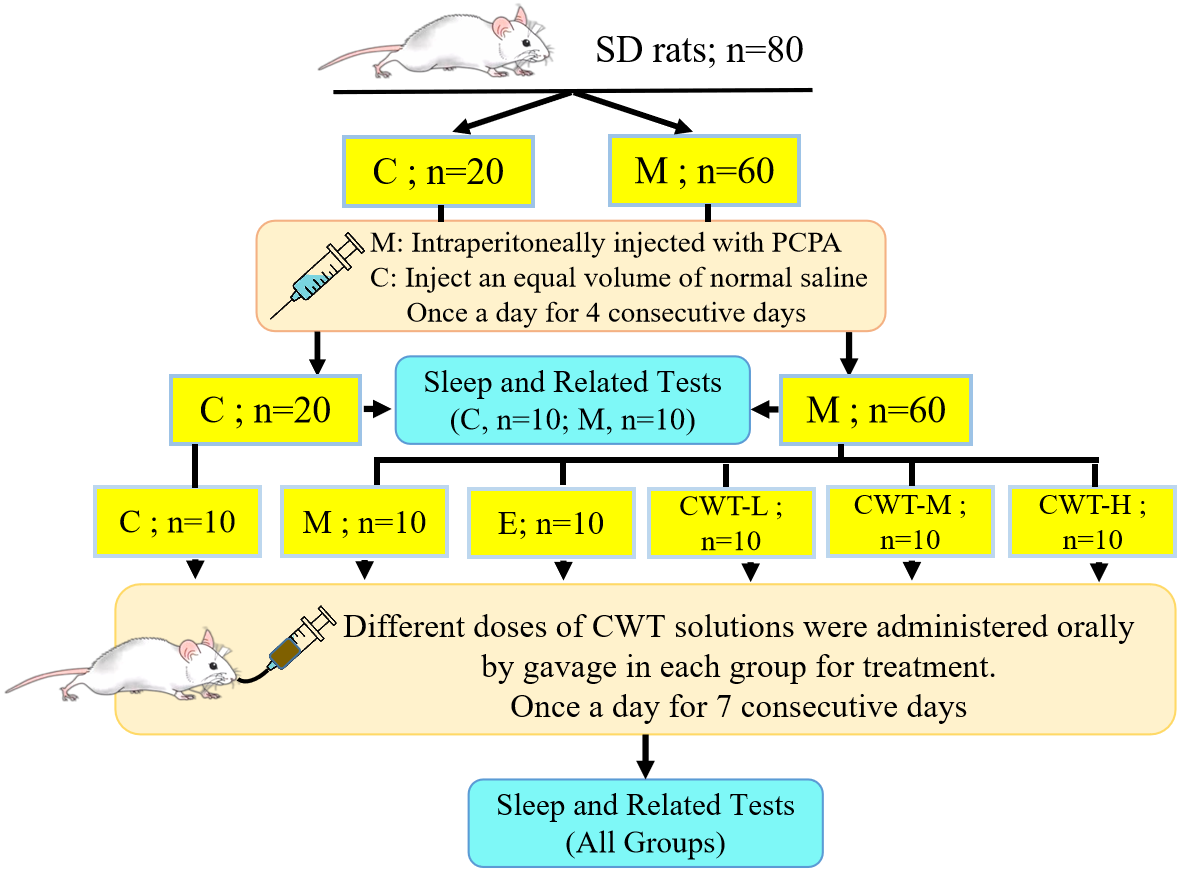


**Supplementary Figure 1.** Flow chart of animal experiment design. (SD rats: Sprague-Dawley rats; C: control group; M: Model group; E: Estazolam group; CWT-L: Ciwujia tablet low dose group; CWT-M: Ciwujia tablet medium dose group; CWT-H: Ciwujia tablet high dose group; PCPA: p-chlorophenylalanine; CWT: Ciwujia Tablet.)

**Supplementary Figure 2.** MS/MS spectrum of chlorogenic acid reference standard solution

**Supplementary Figure 3.** MS/MS spectrum of eleutheroside B reference standard solution

**Supplementary Figure 4.** MS/MS spectrum of eleutheroside E reference standard solution

**Supplementary Figure 5.** MS/MS spectrum of isofraxidin reference standard solution


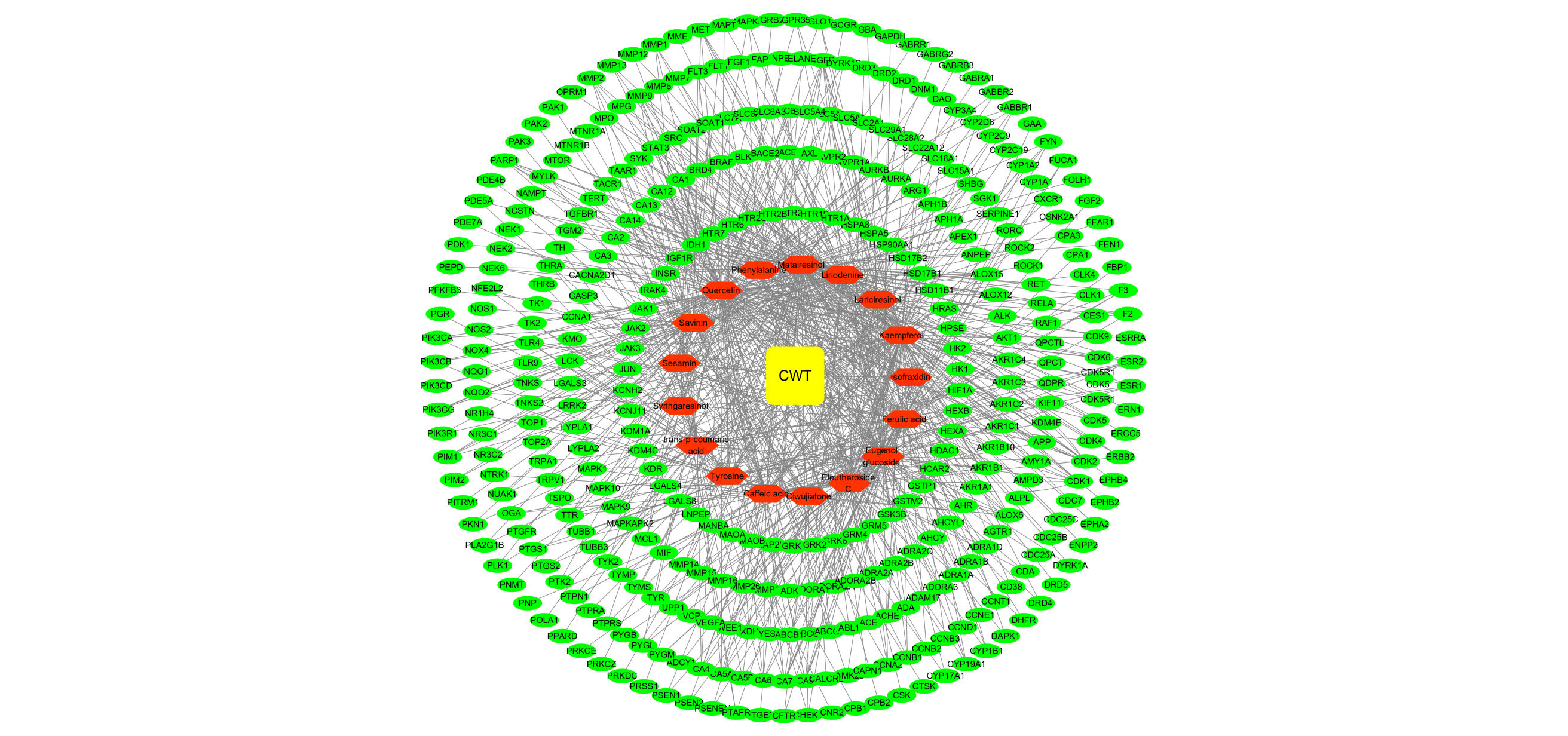
**Supplementary Figure 6.** Ingredient-target Network. The red circles represent the 17 ingredients in CWT; the green circles represent the 377 targets identified in the database.


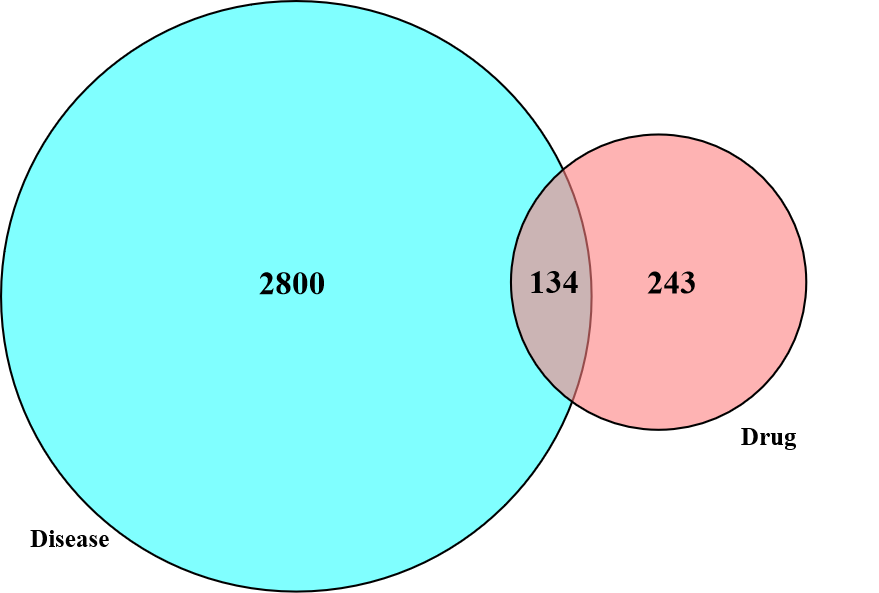


**Supplementary Figure 7.** Venn diagram of 17 ingredient targets related to insomnia


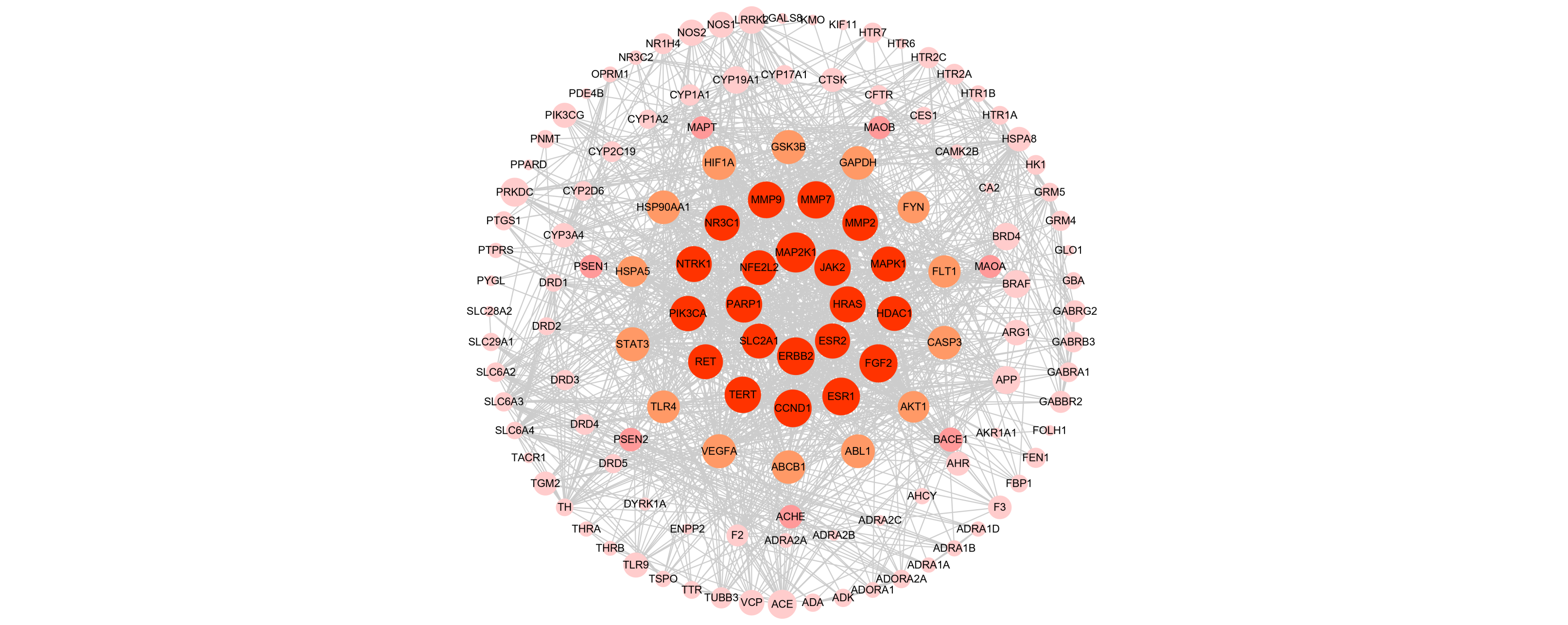


**Supplementary Figure 8.** Anti-insomnia protein target interaction network (Set the size of the circle according to the degree value. The larger the degree value, the larger the circle. The darker the red is, the more important the anti-insomnia effect of this target is).
